# BrainSTEM: A single-cell multiresolution fetal brain atlas reveals transcriptomic fidelity of human midbrain cultures

**DOI:** 10.1126/sciadv.adu7944

**Published:** 2025-10-31

**Authors:** Hilary S. Y. Toh, Lisheng Xu, Carissa Chen, Pengyi Yang, Alfred X. Sun, John F. Ouyang

**Affiliations:** ^1^ Duke-NUS Medical School, Signature Research Program in Neuroscience and Behavioural Disorders, Singapore, Singapore.; ^2^ Computational Systems Biology Unit, Children’s Medical Research Institute, Faculty of Medicine and Health, University of Sydney, Westmead, NSW 2145, Australia.; ^3^ Charles Perkins Centre, School of Mathematics and Statistics, University of Sydney, Sydney, NSW 2006, Australia.; ^4^ Department of Research, National Neuroscience Institute, Singapore, Singapore.; ^5^ Duke-NUS Medical School, Centre for Computational Biology, Singapore, Singapore.; ^6^ Duke-NUS Medical School, Signature Research Program in Cardiovascular and Metabolic Disorders, Singapore, Singapore.

## Abstract

Protocols for deriving midbrain dopaminergic (mDA) neurons for Parkinson’s disease (PD) modeling and therapy remain incompletely benchmarked against in vivo references. To establish transcriptomic standards, we generated an integrated human fetal whole-brain atlas and a midbrain subatlas. Whole-brain analysis revealed strong region-specific signatures, underscoring the need for global mapping before refined midbrain annotation. We implemented this two-tier strategy, BrainSTEM (Brain Single-cell Two tiEr Mapping), to systematically reassess published single-cell datasets of human midbrain culture models. BrainSTEM confirmed the presence of bona fide midbrain cell types (“on-target”), but also revealed substantial populations aligning with nonmidbrain regions (“off-target”), inflating reported mDA yields across protocols. This unbiased framework enables rigorous evaluation of differentiation outcomes, clarifies current limitations of midbrain-directed models, and provides a foundation for refining protocols toward more faithful in vitro systems for PD research and regenerative applications.

## Teaser

Unbiased two-tier mapping to fetal brain single-cell atlases benchmarks regional and cellular heterogeneity in midbrain datasets.

## INTRODUCTION

The human midbrain has been the target of numerous studies due to the selective vulnerability of midbrain dopaminergic (mDA) neurons in Parkinson’s disease (PD) (*1*–*3*). Many mDA neuron differentiation protocols have been proposed (*4*–*6*), modeled after developmental biology principles to pattern stem cell–derived progenitors toward a ventral floor plate fate using small-molecule morphogens (*7*–*10*). However, considerable variation in culture conditions persists, which may complicate the interpretation of disease phenotypes. The heterogeneity in DA neuron yield and uncertain transcriptional fidelity of culture products also poses challenges for PD cell therapy, an active area of clinical trial research (*11*–*13*).

Single-cell RNA (scRNA) sequencing, an emerging technology that can resolve cellular heterogeneity, has been extensively applied to cortical systems (*14*, *15*). While there have been some studies on the developing human midbrain (*16*, *17*), much remains to be explored. In addition, a systematic comparison of in vitro cultures to the actual midbrain is lacking. Furthermore, directly mapping midbrain protocols to a fetal midbrain reference—instead of the whole brain—prematurely limits the scope of the comparison. This can introduce bias as it may not account for the presence of cell types from other brain regions found in midbrain models due to off-target patterning.

In this study, we address these gaps by developing a comprehensive and multiresolution fetal whole-brain atlas and a midbrain subatlas to characterize the molecular fidelity of available midbrain culture datasets. The fetal midbrain subatlas is designed to provide a high-resolution map of the developmental trajectories of midbrain cell types and reveal their interactions. Unlike previous efforts that limit the comparison to specific brain regions, we designed a two-tier projection approach, recognizing that in vitro differentiation often produces off-target cell types and brain regions (*18*). In the first-tier mapping, query datasets are projected onto the fetal whole-brain atlas to assess the brain region identity of various neural populations. Following this, midbrain-specific cells are projected onto the intricately annotated midbrain subatlas. This second higher-resolution projection identifies mDA neurons as well as rare subpopulations that may otherwise be masked by nonmidbrain region cell types. Using this two-tier mapping approach that we termed BrainSTEM (Brain Single-cell Two tiEr Mapping), we conducted an extensive curation of available single-cell datasets, covering both two-dimensional (2D) and 3D organoid models, and examined each protocol’s capacity to recapitulate the in vivo midbrain. Collectively, our work provides a comprehensive resource of the fetal whole brain and midbrain and establishes a reference for evaluating the accuracy of midbrain models for protocol optimization in future development.

## RESULTS

### Integrated whole-brain fetal atlas identifies cell types with brain region–specific gene signatures

**Fig. 1.**
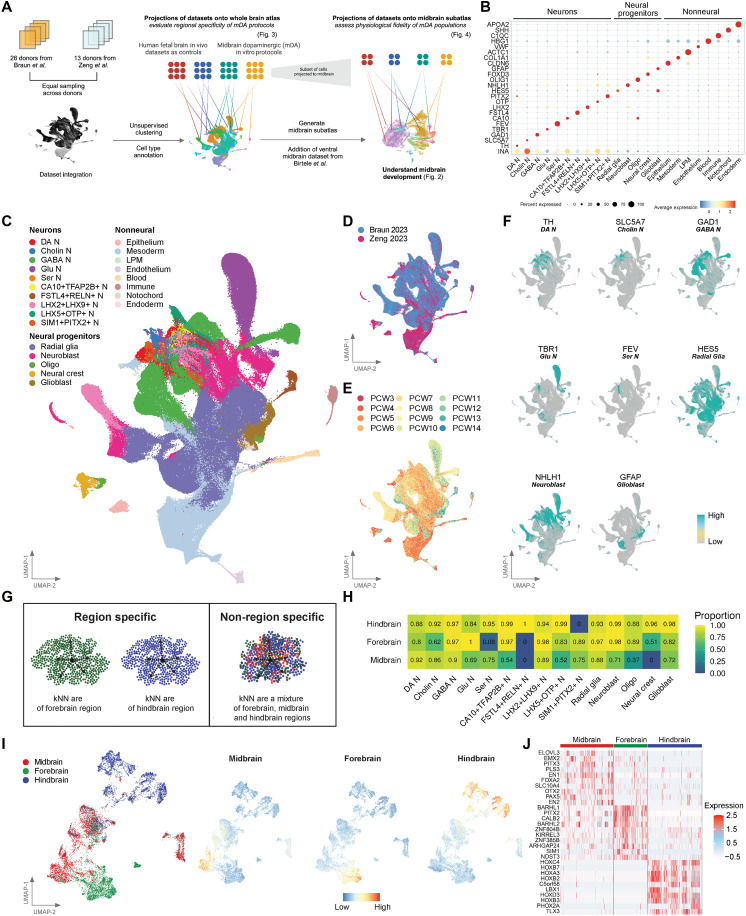
Construction of a fetal whole-brain single-cell atlas identified brain region–specific gene expression signatures across different neural cell types. (**A**) Schematic depicting the computational workflow for constructing the fetal whole-brain single-cell atlas and fetal midbrain subatlas, followed by their downstream applications. This includes projecting in vitro–derived datasets onto these reference atlases to evaluate their transcriptional fidelity. Created in BioRender. Toh, H. (2025); https://BioRender.com/pl47oha. (**B**) Dotplot of gene expression of known marker genes across different annotated cell types in the fetal whole-brain atlas. Uniform Manifold Approximation and Projection (UMAP) plot of fetal whole-brain atlas showing (**C**) the annotated cell types, (**D**) the single-cell study, (**E**) the embryonic age in postconception week, and (**F**) the gene expression of known marker genes for selected cell types. (**G**) Schematic depicting the determination of region specificity by comparing the region identity of each cell’s *k*-nearest neighbors. Created in BioRender. Toh, H. (2025); https://BioRender.com/pl47oha. (**H**) Heatmap showing the average proportion of *k*-nearest neighbors mapping to the corresponding brain region (midbrain, forebrain, or hindbrain) across different cell types. UMAP plot of dopaminergic (DA) neurons showing (**I**) the brain region identity, the midbrain region gene expression signature, the forebrain region gene expression signature, and the hindbrain region gene expression signature. (**J**) Heatmap of gene expression of top 10 marker genes, ordered by log2 fold change, for each brain region in DA neurons.

To create a fetal whole-brain reference atlas containing brain region information, we integrated two datasets by Braun *et al.* (*19*) and Zeng *et al.* (*20*) (Fig. 1A). We leverage on the advantages of both datasets to profile the human brain from postconception week (PCW) 3 to 14 totaling 679,666 cells from 39 donors, providing a highly comprehensive view of early human brain development. Major cell classes from both datasets clustered together and were uniformly distributed (Fig. 1, B to D, and fig. S1, A to C), except for samples from PCW 3 and 4 that were exclusive to Zeng *et al.* (*20*) (Fig. 1E and table S1).

Next, we harmonized cell-type labels across the reference datasets and curated a marker gene panel to annotate the integrated atlas. Our marker gene panel is derived from canonical markers supported by literature and cross-referencing to the original dataset’s annotation criteria (Fig. 1, B and F, and fig. S1D). This consensus annotation approach identified 23 cell types broadly classified as neurons, neural progenitors, and nonneural (Fig. 1B). The neurons comprised 10 cell types, 5 of which were classically defined by neurotransmitters (DA N, Cholin N, GABA N, Glu N, and Ser N) and 5 other cell types labeled with their differentially expressed markers (table S2).

Different brain regions comprise distinct cell types and microenvironments, which manifest as unique transcriptomic signatures. To identify cell types with brain region specificity, we built a *k*-nearest neighbor (kNN) classifier by training it on region labels provided in Braun *et al.*’s (*19*) dataset (Fig. 1G). Focusing on neurons and neural progenitors, cell types with an average classification probability higher than 0.65 were defined to be region specific (Fig. 1H). Of note, we observed high region classification probability for cell types like midbrain DA N (score of 0.92) and forebrain Glu N (score of 1.0), which are known to be distinctive neurons present in the midbrain and forebrain respectively. In contrast, forebrain Ser N were low-scoring (score of 0.08), suggesting a lack of forebrain-specific gene signature for these neurons. As expected, neural progenitor cell types (radial glia, neuroblasts, and glioblasts) displayed region specificity across all three brain regions (fig. S1E), a finding consistent with Braun *et al.* (*19*). The top differentially expressed genes (DEGs) distinguishing forebrain radial glia include well-known genes such as *EMX2* and *FOXG1*, while *EN1* and the *HOX* family of transcription factors mark midbrain and hindbrain radial glia, respectively.

In addition to well-established genes, we also identified markers associated with each region-specific cell type (Fig. 1, I and J, and table S3). Focusing on DA N, we reported 21 genes that specify its midbrain regional identity, including well-known genes such as *EN1* (*21*, *22*) and *PITX3* (*23*) and other less known markers, namely, *ELOVL3* (very long chain fatty acid elongase 3) and *MYRIP* (myosin VIIA and Rab interacting protein). We found little overlap between brain region–specific genes across major neuron subtypes, except for hindbrain, suggesting distinct signatures specifying brain region identity for these neurons (fig. S1F).

### Fetal midbrain subatlas reveals insights into early midbrain development

**Fig. 2.**
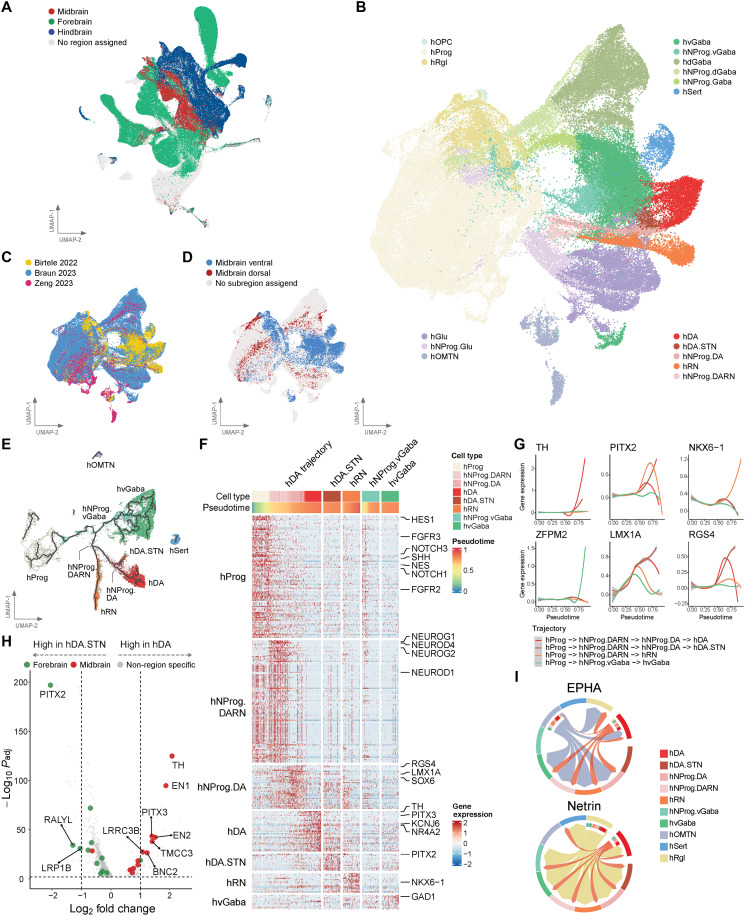
Fetal midbrain subatlas resolved developmental trajectories and rare subpopulations of the early midbrain. (**A**) UMAP plot of fetal whole-brain atlas showing the brain region identity. UMAP plot of fetal midbrain subatlas showing (**B**) the midbrain cell-type annotations, (**C**) the single-cell study, and (**D**) the midbrain subregion, i.e., ventral or dorsal. (**E**) UMAP plot of fetal midbrain ventral subregion-associated cells overlaid with Monocle3-inferred trajectories identifying four major trajectories giving rise to hDA, hDA.STN, hRN, and hvGaba neurons. (**F**) Heatmap showing the gene expression of marker genes for the different cell types present in the four major trajectories identified by Monocle3 algorithm ordered by pseudotime. (**G**) Gene expression of selected genes across pseudotime that are up-regulated in hDA (*TH*), hDA.STN (*PITX2*), hRN (*NKX6-1*), hvGaba (*ZFPM2*), or hNProg.DA (*LMX1A* and *RGS4*). (**H**) Volcano plot showing the differential expression between hDA neurons and hDA.STN neurons. (**I**) Signaling patterns between fetal midbrain ventral subregion-associated cell types for the Ephrin-A (EPHA) and Netrin signaling pathways.

To generate a high-resolution single-cell landscape of the early midbrain, we constructed a midbrain subatlas. We applied our region-based kNN classifier to cells from Zeng *et al.* (*20*) to identify midbrain-associated cells to maximize the number of cells in the subatlas (Figs. 1H and 2A). These cells were then combined with midbrain cells from Braun *et al.* (*19*) and a recently published human ventral midbrain dataset by Birtele *et al.* (*17*). This resulted in a midbrain subatlas comprising 102,335 cells, which is the largest developing human midbrain resource to date, spanning PCW 3 to 14 (Fig. 2, B and C; fig. S2A; and table S4).

To annotate the fetal midbrain subatlas, we adapted the cell-type nomenclature defined by La Manno *et al.* (*16*) in which they reported the first human ventral midbrain dataset, with modifications (fig. S2, B and C). First, we added an “hGlu” cell type found in the dorsal midbrain that was not present in La Manno *et al*.’s (*16*) ventral midbrain. Second, we combined certain cell types such as hRgl1, hRgl2a, hRgl2b, hRgl2c, and hRgl3 into one common label “hRgl” (radial glia). Notably, we introduced previously unidentified midbrain cell-type labels derived from a combination of our trajectory analysis and midbrain subregion information from the reference datasets. These include 6 subtypes of neuronal progenitors [hNProg (hNProg.Glu, hNProg.Gaba, hNProg.vGaba, hNProg.dGaba, hNProg.DARN, and hNProg.DA)] that give rise to their corresponding neurons termed glutamatergic neuron (hGlu), ventral GABA neuron (hvGaba), dorsal GABA neuron (hdGaba), red nucleus (hRN), dopaminergic neuron (hDA), and subthalamic nucleus/dopaminergic neurons (hDA.STN) (Fig. 2B). The cell types hvGaba and hdGaba, and their precursors hNProg.vGaba and hNProg.dGaba are denoted with a “v” or “d” label, highlighting their ventral or dorsal subregion bias based on midbrain subregion metadata (Fig. 2D and fig. S2E). Some cell types such as hDA and hvGaba that reside predominantly in the ventral midbrain subregion contain more cells from Birtele *et al.*’s (*17*) dataset as it is a ventral-only midbrain dataset (fig. S2A). Conversely cell types of a dorsal midbrain identity such as hNProg.dGaba and hdGaba mostly contain cells from Braun *et al.* (*19*) because it is the only parent reference dataset to contain dorsal midbrain tissue (fig. S2A). We further validated the concordance of cell-type labels in the two atlases subatlas and found that major classes of midbrain cell types correspond well to their whole-brain counterpart (fig. S2D). For instance, 97.2% of hDA neurons and 92.2% of hSert neurons in the midbrain subatlas were labeled as DA N and Ser N, respectively, in the whole-brain atlas. Overall, the refined midbrain cell-type annotation corresponds well with the cell-type labels used in the whole-brain atlas (fig. S2D and table S4).

To further delineate the developmental trajectories in the ventral midbrain, we used Monocle3 to perform trajectory inference on ventral-associated cell populations in the fetal midbrain. The inferred trajectories suggest that hvGaba neurons diverge from hRN and hDA earlier in pseudotime (Fig. 2E). hRN, hDA, and hDA.STN neurons appear to share a common progenitor that we labeled as hNProg.DARN (Fig. 2E). Anatomically, these three groups of neurons are in close proximity, supporting the idea that they may originate from a common intermediate progenitor (*24*). From our inferred lineages, hNProg.DARN then gives rise to hRN and hNProg.DA, the immediate precursor to hDA and hDA.STN (Fig. 2E). DEG analysis on these ventral midbrain cell types identified well-known floor plate markers such as *LMX1A* and *SOX6* expressed by hNProg.DA cells and classical dopaminergic marker genes *TH* and *KCNJ6* for hDA (Fig. 2F). We then examined the temporal expression of selected markers for each ventral midbrain neuron subtype. *LMX1A* and *RGS4* are up-regulated in hNProg.DA early in pseudotime and subsequently become down-regulated in hDA but remain highly expressed in hDA.STN (Fig. 2G), suggesting that both nuclei may share a common progenitor but diverge later by differential regulation of certain floor plate progenitor genes.

Detecting hDA.STN in our midbrain subatlas was unexpected, given that the subthalamic nucleus is a diencephalic structure, located in the forebrain (*25*). We confirmed the robustness of this finding as cells from all three studies contributed to this subpopulation, and Braun *et al.* (*19*) have previously reported a similar cell population that they named the PITX2 lineage (*19*). The STN lineage has also been reported to be closely related to the hDA lineage in mouse brain studies (*26*, *27*). hDA.STN and hDA share classical mDA markers like *LMX1A* and *FOXA2*, making it challenging to distinguish in culture systems. We confirmed that *PITX2* is a robust marker of the STN lineage and reported more markers distinguishing these two closely related cell types (Fig. 2H and table S5). Gene set overrepresentation analysis of DEGs revealed a predominance of axonogenesis and dopamine metabolism pathways in hDA neurons, while protein translation– and protein folding–related pathways were up-regulated in hDA.STN neurons (fig. S2F). To understand intercellular communication differences between hDA and hDA.STN, we carried out cell-cell interaction analysis on ventral midbrain cell types. On a global level, hNProg.DARN and hOMTN contribute more to outgoing interactions (fig. S2G). Comparing hDA and hDA.STN, we found the Ephrin-A (EPHA) and Netrin signaling pathways to be specific for hDA (Fig. 2I). These pathways are essential for the formation of dopaminergic neuron circuits (*28*–*30*).

### Projection to whole-brain atlas to identify broad cell types and assess brain region fidelity of midbrain cultures

**Fig. 3.**
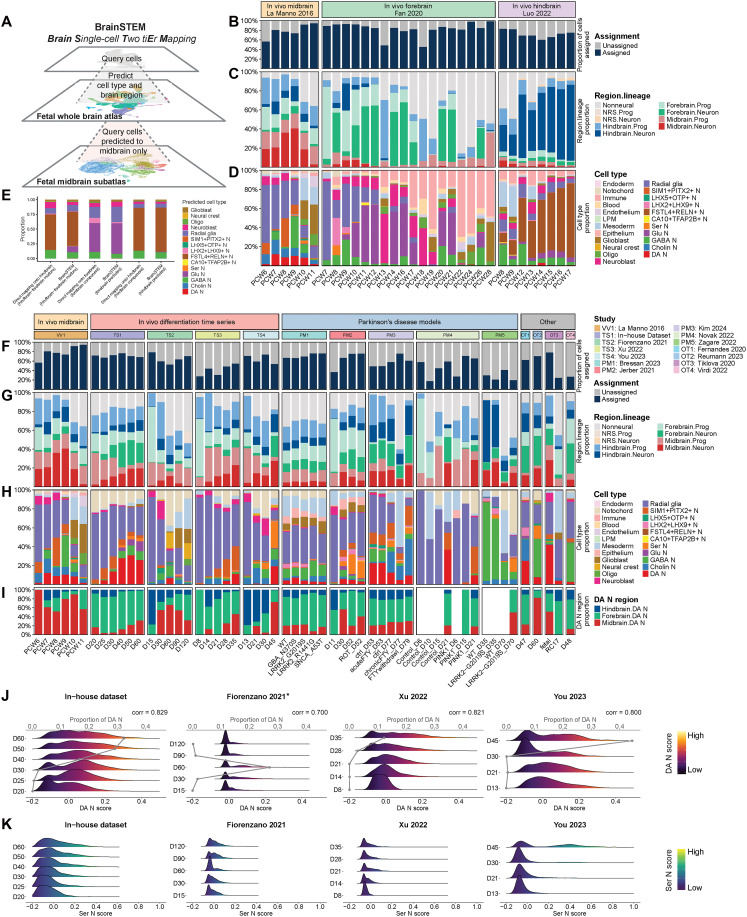
Projection of midbrain culture datasets uncovered differences in cell-type composition and brain region specificity. (**A**) Schematic depicting the BrainSTEM framework’s two-tier mapping process of query datasets onto our fetal whole-brain atlas and midbrain subatlas. Created in BioRender. Toh, H. (2025); https://BioRender.com/pl47oha. Proportion plots of in vivo brain tissue single-cell studies across different developmental stages showing (**B**) proportion of cells confidently assigned, (**C**) region.lineage proportions for confidently assigned cells, and (**D**) cell-type proportions for confidently assigned cells. (**E**) Cell-type proportions of synthetic dataset consisting of 5:1 hindbrain-to-forebrain cells projected either by BrainSTEM or by DM. Proportion plots of in vitro–derived single-cell datasets across different time points and conditions showing (**F**) proportion of cells confidently assigned, (**G**) region.lineage proportions for confidently assigned cells, (**H**) cell-type proportions for confidently assigned cells, and (**I**) brain region proportions for confidently assigned DA neurons. (**J**) Ridge plots showing the distribution of dopaminergic neuron gene expression signature (DA N score) and line graphs showing the proportion of DA N in the midbrain-associated cells across different time points for selected in vitro midbrain differentiation time series studies. Spearman’s correlation coefficient for the DA N score and the proportion of DA N in each study presented. Two-sided Wilcoxon rank sum test applied to assess the pairwise difference between the in-house dataset and the other three datasets, with significance indicated by asterisk [*P* value for the in-house dataset versus Fiorenzano *et al.* (*34*): 0.000, the in-house dataset versus Xu *et al.* (*38*): 0.078, and the in-house dataset versus You *et al.* (*39*): 0.779]. (**K**) Ridge plots showing the distribution of serotonergic neuron gene expression signature (Ser N score) in the hindbrain-associated cells in the same setting.

To determine the cell type and region identity of midbrain cultures, we retrieved published scRNA sequencing (scRNA-seq) data involving midbrain differentiation (*31*–*42*). Overall, we obtained 12 publicly available datasets, spanning both 2D and 3D cultures, and included our in-house midbrain organoid differentiation protocol (*43*), totaling more than 1.4 million cells across 50 conditions (table S6). We grouped them into two categories: (i) in vitro differentiation time series and (ii) PD models. We systematically evaluated each protocol using BrainSTEM, a two-tier mapping framework in which single cells are first projected to the whole-brain atlas and cells with predicted midbrain identity are subsequently mapped to the midbrain subatlas (Fig. 3A). To improve cell-type predictions, we retained confident assignments of projected cell types determined by marker gene module scoring (fig. S3).

We first demonstrated the reliability of whole-brain projection using scRNA-seq datasets of the developing midbrain (*16*), cortex (*44*), and cerebellum (*45*) as controls for the midbrain, forebrain, and hindbrain, respectively. We performed the first-tier projection of these in vivo datasets to the whole-brain atlas and determined their proportion of assigned cell types and projected region identity. All in vivo datasets showed a high percentage of assigned cell types (Fig. 3B) and the majority of their projected regions belonged to their respective brain regions (Fig. 3C). Among the projected cell types, we observed that the midbrain dataset has higher levels of DA neurons compared to datasets from other brain regions, as expected, while the forebrain dataset contains relatively more glutamatergic neurons (Fig. 3D). In the hindbrain dataset, we identified a high proportion of FSTL4+RELN+ neurons. These may correspond to the cerebellar granule neurons that secrete reelin to guide Purkinje cell migration in the hindbrain (*46*).

To mimic the potential regional heterogeneity in culture systems, we generated a synthetic dataset comprising a 5:1 mixture of hindbrain to forebrain cells and subjected it to either BrainSTEM or direct mapping (DM) to a regional reference (Materials and Methods). We found that BrainSTEM mapping recovered Glu N, a cell type predominantly found in the forebrain and was missed by DM to the hindbrain regional reference (Fig. 3E). BrainSTEM also closely recapitulated the 5:1 regional proportions of the synthetic dataset. To further compare the accuracy of predicted cell types for each brain region, we extracted the forebrain and hindbrain components of the synthetic mixture and mapped them to their respective in vivo references. These proportions were highly similar to that predicted by BrainSTEM, suggesting that BrainSTEM can accurately deconvolute the forebrain and hindbrain components of the synthetic mixture and correctly assign their cell types (Fig. 3E). We observed a similar trend for a separate synthetic mixture of 1:5 hindbrain to forebrain cells (fig. S4A). To thoroughly test the ability of BrainSTEM to resolve brain region identity, we tested more synthetic mixtures (1:10 and 1:20) and showed that BrainSTEM was able to recover forebrain- and hindbrain-specific cell populations that DM missed (fig. S4, A to E). Using FSTL4+RELN+ N as a proxy for cells of the hindbrain region, we found increasing proportions of FSTL4+RELN+ N detected by BrainSTEM as the ratio of hindbrain to forebrain cells increased (fig. S4, A and B). Similarly, the proportions of Glu N increased proportionately to the ratio that forebrain cells were combined with hindbrain cells, suggesting that BrainSTEM can quantitatively detect regional impurities (fig. S4, C and D).

Next, we delved into the region identities of confidently assigned cells for each in vitro dataset (Fig. 3F). Notably, cells from forebrain and hindbrain regions constitute more than half of most midbrain datasets (Fig. 3G). We found that our in-house data and the dataset by Xu *et al.* (*38*) displayed the highest yields of midbrain-predicted cells (Fig. 3G). Among the in vitro differentiation time series, midbrain-predicted cells tended to peak between days 30 and 40 in culture. Comparing 2D versus 3D culture conditions, it is not apparent which method is superior in terms of maximizing midbrain region purity. The 3D organoid protocols [in-house data and Fiorenzano *et al.* (*34*)] ranged between 25 and 35% midbrain regional identity at their respective peaks. 2D culture protocols can surpass this, such as the latest time point in Xu *et al.*’s (*38*) dataset, although closer inspection reveals that most of the midbrain cells were composed of progenitors rather than neurons (Fig. 3G).

We next quantified the proportions of projected cell types for each dataset (Fig. 3, D and H). While the transition from radial glia to neuronal cells was observed in most developmental and differentiation time series datasets, the distribution of different neuronal subtypes varied across studies. Benchmarking against La Manno *et al.* (*16*), it is clear that the DA neurons produced in culture are composed of a mixture of regions and their midbrain regional purity does not match that of dissected midbrain tissue (Fig. 3I). Because most midbrain protocols aim to generate mDA neurons and minimize contaminants such as hindbrain serotonergic neurons, we calculated the DA N module score across predicted midbrain cells and the Ser N module score across predicted hindbrain cells to assess the presence of the dopaminergic/serotonergic gene signature in respective regions in the four time series datasets. A two-sided Wilcoxon rank sum test was applied to assess the pairwise difference in DA N module scores between the in-house dataset and the other three studies. The statistical analysis showed that Fiorenzano *et al.* (*34*) reported significantly lower DA N scores than the in-house dataset, while Xu *et al.* (*38*) and You *et al.* (*39*) presented scores similar to those of the in-house dataset. By correlating the average DA N module score with the proportion of predicted DA N cells, we found strong concordance across studies, as indicated by Spearman’s correlation coefficients greater than 0.7 (Fig. 3J). Of note, You *et al.* (*39*) uniquely exhibited a bimodal distribution of serotonergic gene signature with a prominent peak in the positive range at the late time point (day 45), suggestive of excessive caudalization during in vitro patterning in this protocol (Fig. 3K).

### Fetal midbrain subatlas provides high-resolution characterization of midbrain cultures

**Fig. 4.**
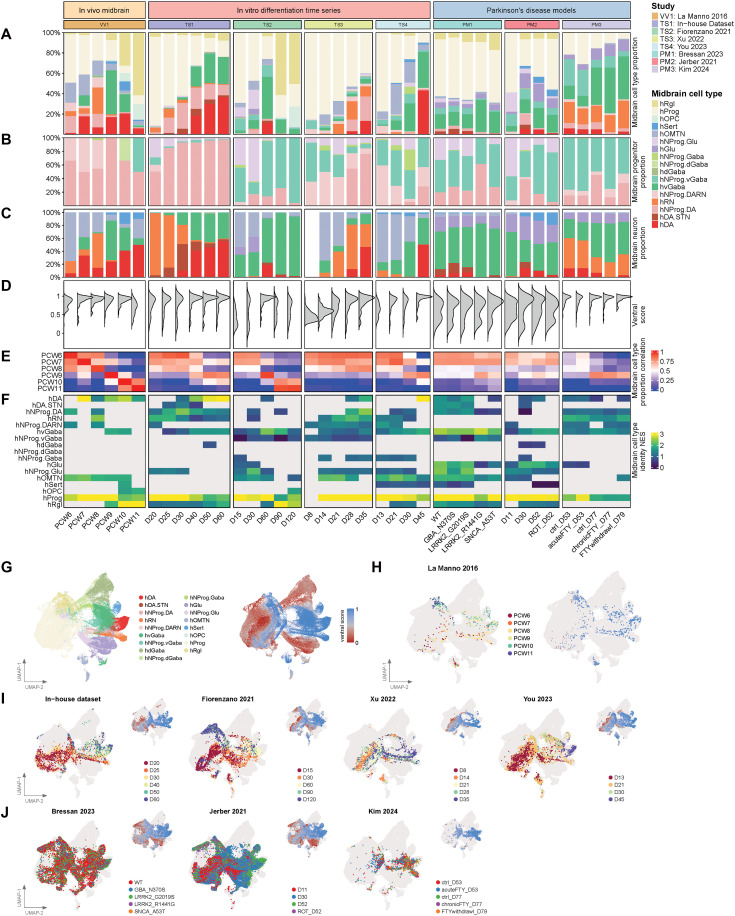
Second-tier projection of midbrain-associated neural cells from midbrain culture datasets revealed differences in cell-type composition, ventral score, and cell-type identity strength. Plots of in vivo and in vitro single-cell studies showing (**A**) midbrain cell-type proportions, (**B**) progenitor proportions, (**C**) neuron proportions, (**D**) distribution of ventral score, (**E**) correlation of midbrain cell-type proportions against in vivo time points, and (**F**) normalized enrichment score of cell-type identity marker genes. (**G**) UMAP plot of fetal midbrain subatlas showing the midbrain cell-type annotations and ventral score. (**H**) UMAP plot of in vivo midbrain data from La Manno *et al.* (*16*) projected onto the fetal midbrain subatlas showing in vivo time points in postconception weeks and ventral score. UMAP plot showing (**I**) different in vitro time series differentiation datasets and (**J**) Parkinson’s disease model datasets projected onto the fetal midbrain subatlas showing their corresponding time points for in vitro differentiation time series and conditions for Parkinson’s disease models. For (I) and (J), the inset UMAP shows the ventral score. For (H) to (J), cells from the fetal midbrain subatlas are plotted in a light gray color to provide context.

Using the BrainSTEM framework, the first-tier projection allows us to extract cells of midbrain identity and subsequently perform a second-tier mapping to the fetal midbrain subatlas (Fig. 3A). With this approach, we are able to map out finer neural cell types introduced in the midbrain subatlas, without confounding cells from other brain regions. Seven in vitro datasets that contained more than 1000 cells after the first-tier projection were mapped onto the fetal midbrain subatlas (Fig. 4A). We evaluated each protocol’s capacity to generate the different neuronal progenitors (hNProg) and neurons of the midbrain. In addition to hNProg.DA, most protocols generated varying proportions of hNProg.vGABA and hNProg.Glu progenitors (Fig. 4B). Correspondingly, hvGABA and hGlu neurons were frequently observed in culture protocols besides hDA neurons (Fig. 4C). We further calculated a ventral score for each dataset (Materials and Methods). Datasets that contained more ventral cell types such as hDA and hNProg.DA (fig. S2E) accordingly received higher ventral scores (Fig. 4D).

To assess how well each protocol recapitulates the cell-type composition of the developing midbrain, we correlated their cell-type proportions to each gestation week of the in vivo midbrain dataset (Fig. 4E). Several datasets displayed high concordance with the developing midbrain from PCW 6 to 8, suggesting that in vitro cultures can recapitulate the cell type diversity during early development. One dataset that cultured midbrain organoids to day 120 showed high correlation to PCW 10 and 11, suggesting that prolonged cultures may indeed mimic later stages of fetal development. To quantify the transcriptomic similarity between in vitro and in vivo counterparts, we calculated cell-type identity scores (Fig. 4F; Materials and Methods). We observed higher cell-type identity scores for progenitor cell types (hProg and hNProg.DA) in earlier time points, while neurons (hDA and hvGaba) scored higher at later time points, consistent with the temporal appearance of these cell types in culture (Fig. 4F). The distribution of projected cell types on the fetal midbrain subatlas’ Uniform Manifold Approximation and Projection (UMAP) space follows the progenitor-to-neuron progression, and their ventral score distribution is shown in Fig. 4 (G to J).

To further ascertain that BrainSTEM’s approach of fine-tuning region produces meaningful improvement in projection outcomes, we carried out DM of several midbrain datasets. We found that the DM approach resulted in elevated numbers of DA neurons (fig. S4C). Closer inspection of these additional DA neurons suggests that they are mainly of forebrain origin based on brain region module score (fig. S4D). Similarly, the regional identity of DA N assigned by DM contains a substantial proportion of forebrain (fig. S4E). These results clearly demonstrate the need for a biologically appropriate reference with sufficient resolution to interrogate cell identity accurately.

In summary, we constructed a fetal whole-brain single-cell atlas and a midbrain subatlas, allowing us to identify cell types with brain region specificity and understand early midbrain developmental trajectories, respectively. This pair of atlases at different resolutions allowed for the establishment of a two-tier projection approach (BrainSTEM) that distinguishes our work from previous efforts to characterize midbrain culture systems. Unlike studies that focus primarily on a single region, BrainSTEM first maps datasets to the entire brain and then refines this by projecting onto a high-resolution midbrain subatlas. This approach allows us to accurately assess the transcriptional fidelity of midbrain cultures, ensuring that both on-target (midbrain) and off-target (nonmidbrain) cell types are considered. With this, we comprehensively evaluated available midbrain differentiation data generated by different protocols from previous studies, highlighting the presence of nonmidbrain contaminants and providing a robust tool for benchmarking. To enable future comparison of midbrain differentiation protocols, we provide an easy-to-use R package (https://github.com/the-ouyang-lab/BrainSTEM) for users to map their own single-cell datasets. Furthermore, we present the fetal whole-brain and midbrain atlases as an online resource (http://brainstem.ouyanglab.com/).

## DISCUSSION

In this study, we present the integration of two large-scale developing human whole-brain datasets. Incorporating the dataset by Zeng *et al.* (*20*) expanded the developmental age range of our atlas and harmonizing cell-type labels from both studies resulted in better coverage of neuronal subtypes than each original dataset. This integrated brain atlas allowed for the systematic identification of region-specific gene signatures and demonstrated the variation in brain region specificity within key neural cell types.

A central contribution of this work involves the generation of the largest integrated human fetal midbrain resource. The fetal midbrain subatlas demonstrates neuronal progenitor cell types we identified through trajectory analysis. Of note, we identified a small subpopulation of hDA (termed hDA.STN) bearing transcriptomic similarity to neurons of the subthalamic nucleus that are known to share common markers with dopaminergic neuron lineage. Detecting hDA.STN in the midbrain subatlas suggests that our atlas can resolve rare cell types, given that it constitutes less than 1% in terms of cell numbers of the midbrain subatlas. As the ventral midbrain is the initial site of DA neuron degeneration in PD, many midbrain differentiation protocols strive to generate cultures of high ventral identity. Thus, we investigated the developmental trajectories and cell-cell communication of the ventral midbrain subregion in more detail. Through this, we identified genes that may distinguish hDA and hRN neurons that originate from the floor plate and basal plate, respectively. These findings offer new insights into the regulatory networks required for patterning within the midbrain.

Using the atlases we curated, we performed a comprehensive evaluation of available midbrain differentiation protocols using the BrainSTEM approach, revealing a wide range of cell types and proportions that constitute each dataset. Rare cell populations such as hDA.STN could now be resolved with the second-tier mapping. While we observed a general trend toward maturation over time, the temporal development of specific cell types varies among protocols. While each dataset uses different culture methods, we observed that protocols that included FGF8 and used relatively lower doses of CHIR yielded more dopaminergic neurons (Fig. 4 and table S6). Our work provides a starting point for inferring morphogen effects on midbrain differentiation to enrich specific cell types like mDA neurons, useful for improving PD cell therapy products.

As neural culture systems advance, mapping these biologically complex in vitro products should reflect the diversity of cell types, regions, and even sub-regions it may contain. Tools to interrogate brain region identity in neural culture models, such as VoxHunt, have been developed in recent years (*47*). Atlas-level resources that contain spatial information available for mapping neural models are often based on the mouse brain (*48*–*50*). More recently, a repository of nonhuman primate multiomics brain data was published (*51*). While useful for cross-species comparison, these tools do not address the gap of characterizing human-specific regional brain features of neural culture models.

By projecting midbrain datasets to a human whole-brain atlas first, BrainSTEM does not prematurely limit the scope of the cell populations that can be mapped. In contrast to conventional approaches that project midbrain datasets directly to a midbrain reference, BrainSTEM can exclude more off-target regions (fig. S4). As compared to DM, BrainSTEM results consistently score higher in regional purity and identifies cell types specific to particular regions. We demonstrated this in all major brain regions using both in vivo and in vitro datasets, showing that BrainSTEM effectively models regional signatures across neural cell types. The differences, while subtle, underscore the importance of accounting for regional brain identity, which may influence a cell type’s functional and maturation profile. When projecting single-cell data for disease modeling, nuanced differences may contribute to an overall disease phenotype that methods like BrainSTEM are primed to detect. Extracting only hDA cells from the dataset by Jerber *et al.* (*33*), we illustrated that this targeted analysis was able to uncover an enrichment of microglia-related terms in rotenone-treated samples suggestive of neuroinflammation in the disease model (fig. S3B). Projection of single-cell transcriptomic data to large reference atlases can be computationally intensive, and conventional analyses to determine cell types by DM may be sufficient to identify one’s target cell population such as DA neurons. Hence, we designed our BrainSTEM as a ready-to-use R package that makes reference mapping accessible to most users. The advantages that we have demonstrated with BrainSTEM’s two-tiered mapping are substantial, especially for applications that require high-purity cell products. For instance, manufacturing of good manufacturing practice (GMP)–grade cells for PD cell replacement therapy requires a well-characterized population of cells that will both meet safety standards and deliver therapeutic outcomes. Using a framework like BrainSTEM, future protocols can be rigorously assessed to improve brain regional purity. Beyond the midbrain, we envision that the BrainSTEM framework will pioneer the importance of multitier mapping to account for different cellular resolutions in complex biological systems.

One important limitation of this study is the developmental time window that we have access to. The reference atlases span only the first trimester, and time points may have unequal coverage due to limited access to human fetal samples. The in vivo forebrain and hindbrain datasets used as validation for the BrainSTEM approach also extend beyond the first trimester. We note a corresponding decrease in accurate regional identity predictions, especially for the forebrain dataset, after PCW 17, suggesting regional maturation transcriptional signatures that are not captured by the reference atlas. Although our atlases are well-positioned to profile early stages of DA neuron development, these neurons continue to mature and form functional circuits beyond the first trimester, thus limiting our assessment of the maturation profile of in vitro midbrain cultures (*52*, *53*). Although our integrated whole-brain atlas contains cell types labeled as Glioblast and Oligo, mature glial cell types such as astrocytes and oligodendrocytes are not a major constituent in the current atlas as they develop and mature after the first trimester (*54*). Thus, extending the time frame of the reference atlases may offer substantial insights into cell-cell communication that takes place in the developing brain.

Increasing evidence supports the unique gene expression identity underlying regional differences among neural cell types. We have also identified putative marker genes acting in the midbrain for subregion specification. These marker genes are subject to experimental validation to confirm their spatial distribution and temporal expression during development. Through our survey of available midbrain differentiation protocols, we observed that each protocol can recapitulate some but not all aspects of the in vivo midbrain. Our fidelity metrics are certainly not exhaustive, and future studies may consider profiling the epigenetic and spatial transcriptomic landscape of in vitro protocols with the emergence of multimodal single-cell technologies. Nonetheless, the fetal midbrain subatlas represents a unified single-cell resource of the midbrain, which can contribute to the understanding of midbrain development in vivo and aid in refining midbrain protocols to advance the study of PD pathogenesis and cell therapy.

## MATERIALS AND METHODS

### Metadata curation and preprocessing of human fetal whole-brain scRNA-seq datasets

We used two recently published fetal brain datasets, Braun *et al.* (*19*) and Zeng *et al.* (*20*), to generate our human fetal whole-brain atlas. These two reference datasets together span PCW 3 to 14 of gestation contributed by 39 donors. We sampled an equal number of cells (*n* = 17,665 cells) from each donor, except for two donors that had fewer cells than the rest; hence, all cells were used from those two donors. All preprocessing and analysis were carried in Seurat (v4.9.9) unless indicated otherwise. Each down-sampled dataset was log-normalized using the “NormalizeData” function, highly variable features were extracted using the “FindVariableFeatures” function and scaled using the “ScaleData” function. Variance rank in each dataset was combined, and we determined the top 3000 highly variable features from the combined rank. The consensus highly variable features were used for downstream analysis. We constructed the human fetal whole-brain atlas by integrating the datasets using the Seurat anchor-based reciprocal principal component analysis (RPCA) method (*55*). Briefly, batch effect correction was performed using the 13 donor identifiers from Zeng *et al.* (*20*) and on the 10x Chromium chemistry version (v2 or v3) from Braun *et al.* (*19*) as the batches. Each batch was log-normalized and scaled, and PCA was calculated on the top 3000 consensus highly variable features. Integration anchors were identified with the Seurat “FindIntegrationAnchors” function and the top 50 PCs were used to integrate the datasets using the Seurat “IntegrateData” function, specifying the Seurat RPCA method.

### Cell-type annotation of the human fetal whole-brain atlas

Unsupervised clustering was performed using Seurat’s “FindNeighbors” and “FindClusters” functions using the top 50 PCs and a clustering resolution of 2.0. To annotate the clusters, we first harmonized cell-type labels from the two reference datasets that compose our human fetal whole-brain atlas. Matching cell types present in both datasets (e.g., “blood” and “erythrocyte”) were combined into the same label. Cell types present in only one of the datasets were retained [e.g., “Oligo” in Braun *et al.* (*19*)]. For neurons, we kept the subtype labeling by Zeng *et al.* (*20*) (“Cholin N,” “DA N,” “GABA N,” and “Glu N”) and added one more category for serotonergic neurons. All major neural classes are represented in the final cell-type annotation, except for microglia and pericytes, which are found in the “Immune” and “Mesoderm” clusters, respectively. To maintain consistency with the naming conventions of the parent datasets, they were not relabeled during our annotation. A panel of marker genes informed by literature and databases for each cell type was used to annotate the atlas. To refine the clustering, we performed subclustering for selected cell types and benchmarked the annotation against our marker gene panel.

### kNN classifier to identify cell types with distinct transcriptomic profiles across brain regions and assign region identity for these cell types

Brain region metadata are available in the Braun *et al.* (*19*) dataset, but not in the Zeng *et al.* (*20*) dataset. To determine the brain region specificity for each cell type, we trained a kNN classifier with *k* = 10 on the top 50 PCs for each cell type to predict the brain region identity (midbrain/forebrain/hindbrain) at the single-cell level, using the R package class (v7.3). The kNN classifier was trained on cells from Braun *et al.* (*19*), leveraging on the available brain region metadata. Region-specific cell types were defined as those with an average classification probability higher than 0.65 across all cells in that cell type. This indicates that most of the cell’s kNNs belong to the same brain region, implying distinct transcriptomic differences between brain regions for that particular cell type. As expected, all nonneural cell types have low classification probability. These cell types, together with neural cell types with classification probability lower than 0.65, were labeled as non–region specific. For the remaining region-specific neural cell types, the kNN classifier was applied to cells from Zeng *et al.* (*20*) to infer their brain region identity.

### Generation of the human fetal midbrain subatlas

The fetal midbrain subatlas comprises three reference datasets. First, from the Braun *et al.* (*19*) reference dataset, we subset all neural cells from the midbrain region using the region metadata provided. Second, from the Zeng *et al.* (*20*) dataset, we used the kNN classifier to infer region identity for the 10 cell types that show midbrain region specificity, namely, Cholin N, DA N, GABA N, Glu N, Ser N, LHX2+LHX9+ N, SIM1+PITX2+ N, radial glia, neuroblast, and glioblast. From these cells, we kept those with inferred midbrain region identity. Last, we included a third reference human ventral midbrain dataset published by Birtele *et al.* (*17*) (excluding nonneural cells). Each dataset was log-normalized and scaled separately before integration using the Seurat RPCA approach. Top 3000 consensus highly variable genes were calculated by “SelectIntegrationFeatures.” All other parameters were the same as those in the fetal whole-brain atlas construction.

### Cell-type annotation of the human fetal midbrain subatlas

To annotate the integrated fetal midbrain subatlas, we first performed unsupervised clustering using Seurat’s FindNeighbors and FindClusters functions applied to the top 50 PCs with a clustering resolution of 3.0. We derived the midbrain marker gene annotation panel from La Manno *et al.*’s (*16*) dataset and computed module scores for the top 10 markers of each cell type. Clusters were manually annotated based on the strength of module score expression. Acknowledging the transcriptional differences among neuronal progenitors (hNProg), we subset the hNProg cells with each of the terminal neuron population and generated UMAPs to visualize the relationship between hNProg and their corresponding neurons. We then attached finer labels of hNProg subtypes to selected clusters based on their proximity to the corresponding neurons.

### Identification of DEGs

DEGs were computed using Seurat’s “FindAllMarkers” function in several analyses, namely, (i) identification of cell type–specific genes for all the cell types in the fetal whole-brain atlas, (ii) identification of brain region–specific genes across the 10 cell types that show midbrain region specificity, (iii) identification of cell type–specific genes for all the cell types in the fetal midbrain subatlas, (iv) identification of cell type–specific genes across selected cell types in the La Manno *et al.* (*16*) dataset, (v) identification of cell type–specific genes for all the cell types in the ventral subregion subset of the fetal midbrain subatlas and using Seurat’s “FindMarkers”, and (vi) identification of cell type–specific genes between hDA and hDA.STN. Genes with average log fold change >1.5, false discovery rate <0.01 were defined as highly expressed for that cluster.

### Reconstruction of developmental trajectories within fetal midbrain subatlas

As we are mainly interested in the developmental trajectories in the ventral subregion of the fetal midbrain, we further subsetted clusters with a median ventral score of >0.5 across all the fetal midbrain cell types. This excluded dorsal-specific cell types, e.g., hGlu and hdGaba. For the resulting ventral subregion subset, the top 50 PCs were recalculated and then used to generate UMAP coordinates. To reconstruct the developmental trajectories, Monocle3 (*56*) (v1.3.7) was applied to the UMAP coordinates, identifying four major branches in the main trajectory, ending with the hDA, hDA.STN, hRN, and hvGaba cell types, respectively. To calculate the pseudotime, diffusion pseudotime from scanpy package (v1.10.1) was used, setting the single cell in cluster 36 (which has the lowest median ventral score) that is furthest away from all other clusters as the root cell, i.e., the cell with zero pseudotime. The top marker genes identified using Seurat’s FindAllMarkers function were then visualized across the four different branches along pseudotime. Furthermore, the gene expression pattern of selected genes across the four different branches along pseudotime is interpolated using the loess function in R.

### Cell-cell interaction analysis

Cell-cell communication signaling pathways among the ventral midbrain cell types were analyzed against the curated repositories of ligand-receptor interactions from CellChatDB (*57*) (v1 and v2) and CellPhoneDB (*58*) (v4.1). Communication probabilities accounting for sample variability using the default pipeline and interactions found in at least one study were kept. The “netAnalysis_signalingRole_heatmap” function in CellChat was used to visualize the relative signaling strengths for all signaling pathways. The “netVisual_chord_cell” function was used to visualize the relative signaling strengths between interacting cell types of selected pathways that are specific to hDA or hDA.STN cell type.

### hPSC culture and generation of in-house midbrain organoids

Human embryonic stem cells (WiCell, WA09) were maintained feeder-free on Matrigel (Corning, #354277)–coated plates in TeSR-E8 medium (Stem Cell Technologies, #05990). Human embryonic stem cells (hESCs) were dissociated into single cells with TrypLE (Gibco, #12604021). A total of 6000 cells per well were seeded into 96-well low-attachment plates to form embryoid bodies overnight. Generation of in-house midbrain organoids was performed as described in (*43*) with some modifications: (i) replacing Noggin with 100 nM LDN193189 (SelleckChem, #S2618), and (ii) replacing SHH with 0.5 μM purmorphamine (Tocris, #4551) Organoids were embedded in Matrigel droplets on day 6 and shifted to spinner culture from day 8 onward. For long-term maturation, the organoids were maintained in media containing MACS Neuro medium (Miltenyi Biotec, #130-093-570) with NeuroCult SM1 without vitamin A (Stem Cell Technologies) supplemented with Brain-Derived Neurotrophic Factor (BDNF) (10 ng/ml; Stem Cell Technologies, #78005.2), Glial cell line-Derived Neurotrophic Factor (GDNF) (10 ng/ml; Stem Cell Technologies, #78058.2), and 100 μM db-cAMP (Sigma-Aldrich, #D0627), and cultured using an orbital shaker. Medium was refreshed every 3 days.

### Library preparation, sequencing, and raw data processing

Midbrain organoids were dissociated into single cells using Papain-based dissociation. Samples were fixed and stored for scRNA-seq according to the Parse Biosciences Evercode Fixation kit (v2.0.2). Library preparation was conducted using the Parse Biosciences Evercode Whole Transcriptome (WT) kit (v2.0.1). Sequencing sublibraries were quantified with the KAPA Library Quantification Kit (#KK4824). Sequencing was performed on the Illumina NovaSeq 6000 platform. Sequencing raw reads were demultiplexed and processed using the Parse Biosciences pipeline (v1.0.5), aligned to the GrCH38 human genome. Cells with at least 800 genes detected and less than 15% mitochondrial reads were retained for analysis.

### Projection of query datasets to the fetal brain reference atlases

To compare midbrain differentiation protocols to the fetal whole-brain reference, we used Seurat’s “FindTransferAnchors” with RPCA approach, followed by Seurat’s “MapQuery” function, a wrapper function of “TransferData,” “IntegrateEmbeddings,” and “ProjectUMAP” to obtain predicted cell-type identities of each query and their projected coordinates on the reference UMAP. Seurat (v5.0.2) was used and the reference Seurat objects were updated to Seurat v5 format.

All query datasets were downloaded from their respective sources (table S6) and used as it is, except for the following: (i) for (*33*), the day 52 time point was downsampled by half, and each time point was mapped separately; (ii) for (*35*), induced pluripotent stem cell samples were removed before mapping; and (iii) for (*41*), striatal and cortical cells were removed before mapping. For each query, we calculated a module score for each predicted cell type using the whole-brain reference’s top 20 marker genes. Cells with a module score >0 for their predicted cell type were retained as confidently assigned cells. Next, to assign region identity, we plotted the module score of each regional cell type’s top 20 marker genes. The region with the highest module score is taken to be the predicted region of the query cell. After the first projection to the fetal whole-brain atlas, we extracted the midbrain region of each query dataset. Datasets containing fewer than 1000 midbrain cells were discarded. The midbrain cells of the remaining datasets were projected to the fetal midbrain subatlas using the same projection as described above.

### Comparison of query datasets

To compare query datasets after the first projection to the whole brain, we computed the (i) proportion of cells confidently assigned, (ii) region.lineage proportions for confidently assigned cells, (iii) cell-type proportions for confidently assigned cells, and (iv) brain region proportions for confidently assigned DA neuron cells. To compare query datasets after the second projection to the midbrain subatlas, we computed the (i) midbrain cell-type proportions, (ii) progenitor proportions, (iii) neuron proportions, (iv) distribution of ventral score, (v) correlation of midbrain cell-type proportions against in vivo time points, and (vi) normalized enrichment score of cell-type identity marker genes. For ventral score calculation, see the next section. To correlate the midbrain cell-type proportions, we first calculated the midbrain cell-type proportions for each time point of the in vivo midbrain dataset [La Manno *et al.* (*16*)] spanning PCW 6 to 11. The Pearson correlation was then calculated between each of these in vivo time points and the cell-type proportions for confidently assigned cells in each time point/condition of the query datasets. To assess the transcriptional robustness of the assigned cells, we first calculated the log2 fold change for all genes comparing the predicted cell type of interest against all other predicted cell types in the query dataset. This log2 fold change was then used as an input for Gene Set Enrichment Analysis using the top 50 marker genes for the predicted cell type of interest as the gene set. The resulting Normalized Enrichment Score reflects how well the up-regulated genes in the predicted cell type of interest are enriched for the marker gene set.

### Ventral score classification

The R package ranger (v0.15.1) was used to train a random forest classifier on midbrain ventral and dorsal subregion differences based on labels provided by the Braun *et al.* (*19*) dataset and the Birtele *et al.* (*17*) dataset. The model was used to predict a score for each query at a single-cell level, with higher scores corresponding to higher ventral identity. The resulting ventral score was visualized on UMAP using Seurat’s “FeaturePlot” function.

### DM of query datasets for comparison to BrainSTEM

In the BrainSTEM framework, we adopted a two-tier mapping strategy whereas the conventional projection approach maps query datasets directly onto a regional reference, which we call DM in this study. To demonstrate that BrainSTEM accurately resolves regional identity, we created synthetic datasets consisting of (a) a 5:1 mixture of hindbrain cells (*45*) and forebrain cells (*44*) and (b) a 1:5 mixture of hindbrain cells (*45*) and forebrain cells (*44*) from in vivo datasets where regional identity is known. These synthetic mixtures thus mimic culture conditions whereby cells of multiple regions may exist. To carry out DM of these synthetic datasets, we prepared a forebrain and hindbrain reference derived from (*19*). We performed the following mapping conditions: (i) DM of the synthetic dataset to majority region reference [i.e., synthetic dataset (a) is mapped to the hindbrain reference and synthetic dataset (b) is mapped to the forebrain reference], (ii) BrainSTEM mapping of the synthetic dataset, (iii) DM of the forebrain component of the synthetic dataset to the forebrain reference, and (iv) DM of the hindbrain component of the synthetic dataset to the hindbrain reference.

To specifically compare mapping outcomes for DA neurons, we prepared a midbrain reference for DM that would (i) best represent what users will use as a reference in the absence of our midbrain subatlas and (ii) allow for comparison of the projection results between BrainSTEM and DM. The midbrain subset derived from Braun *et al.*’s (*19*) dataset was used as the reference for DM as it is the current largest fetal midbrain dataset from a single study. Furthermore, the cells in this DM reference exist in both the fetal whole-brain atlas and the fetal midbrain subatlas; thus, the DM reference contains cell-type labels from both. We directly mapped the in-house dataset, together with selected in vivo and in vitro midbrain datasets to the DM reference, to emphasize the varied regional proportions of DA neurons as compared to the BrainSTEM framework. FindTransferAnchors with RPCA approach and MapQuery functions from Seurat (v5.0.2) were used for DM as previously described.

## References

[1] A. Samii, J. G. Nutt, B. R. Ransom, Parkinson’s disease. Lancet 363, 1783–1793 (2004). 15172778 10.1016/S0140-6736(04)16305-8

[2] S. A. Jagmag, N. Tripathi, S. D. Shukla, S. Maiti, S. Khurana, Evaluation of models of Parkinson’s disease. Front. Neurosci. 9, 503 (2015). 26834536 10.3389/fnins.2015.00503 PMC4718050

[3] S. J. Chia, E. K. Tan, Y. X. Chao, Historical perspective: Models of Parkinson’s disease. Int. J. Mol. Sci. 21, 2464 (2020). 32252301 10.3390/ijms21072464 PMC7177377

[4] S. Kriks, J. W. Shim, J. Piao, Y. M. Ganat, D. R. Wakeman, Z. Xie, L. Carrillo-Reid, G. Auyeung, C. Antonacci, A. Buch, L. Yang, M. F. Beal, D. J. Surmeier, J. H. Kordower, V. Tabar, L. Studer, Dopamine neurons derived from human ES cells efficiently engraft in animal models of Parkinson’s disease. Nature 480, 547–551 (2011). 22056989 10.1038/nature10648 PMC3245796

[5] A. Kirkeby, S. Grealish, D. A. Wolf, J. Nelander, J. Wood, M. Lundblad, O. Lindvall, M. Parmar, Generation of regionally specified neural progenitors and functional neurons from human embryonic stem cells under defined conditions. Cell Rep. 1, 703–714 (2012). 22813745 10.1016/j.celrep.2012.04.009

[6] J. Xi, Y. Liu, H. Liu, H. Chen, M. E. Emborg, S. C. Zhang, Specification of midbrain dopamine neurons from primate pluripotent stem cells. Stem Cells 30, 1655–1663 (2012). 22696177 10.1002/stem.1152 PMC3405174

[7] E. Andersson, U. Tryggvason, Q. Deng, S. Friling, Z. Alekseenko, B. Robert, T. Perlmann, J. Ericson, Identification of intrinsic determinants of midbrain dopamine neurons. Cell 124, 393–405 (2006). 16439212 10.1016/j.cell.2005.10.037

[8] A. L. M. Ferri, W. Lin, Y. E. Mavromatakis, J. C. Wang, H. Sasaki, J. A. Whitsett, S.-L. Ang, Foxa1 and Foxa2 regulate multiple phases of midbrain dopaminergic neuron development in a dosage-dependent manner. Development 134, 2761–2769 (2007). 17596284 10.1242/dev.000141

[9] M. Wang, K.-H. Ling, J. J. Tan, C.-B. Lu, Development and differentiation of midbrain dopaminergic neuron: From bench to bedside. Cells 9, 1489 (2020). 32570916 10.3390/cells9061489 PMC7349799

[10] H. S. Y. Toh, X. Y. Choo, A. X. Sun, Midbrain organoids—Development and applications in Parkinson’s disease. Oxf Open Neurosci. 2, kvad009 (2023). 38596240 10.1093/oons/kvad009 PMC10913847

[11] A. Kirkeby, M. Parmar, R. A. Barker, Strategies for bringing stem cell-derived dopamine neurons to the clinic: A European approach (STEM-PD). Prog. Brain Res. 230, 165–190 (2017). 28552228 10.1016/bs.pbr.2016.11.011

[12] L. Studer, Strategies for bringing stem cell-derived dopamine neurons to the clinic—The NYSTEM trial. Prog. Brain Res. 230, 191–212 (2017). 28552229 10.1016/bs.pbr.2017.02.008

[13] J. Takahashi, Strategies for bringing stem cell-derived dopamine neurons to the clinic: The Kyoto trial. Prog. Brain Res. 230, 213–226 (2017). 28552230 10.1016/bs.pbr.2016.11.004

[14] S. Kanton, M. J. Boyle, Z. He, M. Santel, A. Weigert, F. Sanchis-Calleja, P. Guijarro, L. Sidow, J. S. Fleck, D. Han, Z. Qian, M. Heide, W. B. Huttner, P. Khaitovich, S. Paabo, B. Treutlein, J. G. Camp, Organoid single-cell genomic atlas uncovers human-specific features of brain development. Nature 574, 418–422 (2019). 31619793 10.1038/s41586-019-1654-9

[15] A. Uzquiano, A. J. Kedaigle, M. Pigoni, B. Paulsen, X. Adiconis, K. Kim, T. Faits, S. Nagaraja, N. Anton-Bolanos, C. Gerhardinger, A. Tucewicz, E. Murray, X. Jin, J. Buenrostro, F. Chen, S. Velasco, A. Regev, J. Z. Levin, P. Arlotta, Proper acquisition of cell class identity in organoids allows definition of fate specification programs of the human cerebral cortex. Cell 185, 3770–3788.e27 (2022). 36179669 10.1016/j.cell.2022.09.010 PMC9990683

[16] G. La Manno, D. Gyllborg, S. Codeluppi, K. Nishimura, C. Salto, A. Zeisel, L. E. Borm, S. R. W. Stott, E. M. Toledo, J. C. Villaescusa, P. Lonnerberg, J. Ryge, R. A. Barker, E. Arenas, S. Linnarsson, Molecular diversity of midbrain development in mouse, human, and stem cells. Cell 167, 566–580.e19 (2016). 27716510 10.1016/j.cell.2016.09.027 PMC5055122

[17] M. Birtele, P. Storm, Y. Sharma, J. Kajtez, J. N. Wahlestedt, E. Sozzi, F. Nilsson, S. Stott, X. L. He, B. Mattsson, D. R. Ottosson, R. A. Barker, A. Fiorenzano, M. Parmar, Single-cell transcriptional and functional analysis of dopaminergic neurons in organoid-like cultures derived from human fetal midbrain. Development 149, dev200504 (2022). 36305490 10.1242/dev.200504 PMC10114107

[18] H. J. Kim, M. O’Hara-Wright, D. Kim, T. H. Loi, B. Y. Lim, R. V. Jamieson, A. Gonzalez-Cordero, P. Yang, Comprehensive characterization of fetal and mature retinal cell identity to assess the fidelity of retinal organoids. Stem Cell Rep. 18, 175–189 (2023). 10.1016/j.stemcr.2022.12.002 PMC9860116 36630901

[19] E. Braun, M. Danan-Gotthold, L. E. Borm, K. W. Lee, E. Vinsland, P. Lonnerberg, L. Hu, X. Li, X. He, Z. Andrusivova, J. Lundeberg, R. A. Barker, E. Arenas, E. Sundstrom, S. Linnarsson, Comprehensive cell atlas of the first-trimester developing human brain. Science 382, eadf1226 (2023). 37824650 10.1126/science.adf1226

[20] B. Zeng, Z. Liu, Y. Lu, S. Zhong, S. Qin, L. Huang, Y. Zeng, Z. Li, H. Dong, Y. Shi, J. Yang, Y. Dai, Q. Ma, L. Sun, L. Bian, D. Han, Y. Chen, X. Qiu, W. Wang, O. Marin, Q. Wu, Y. Wang, X. Wang, The single-cell and spatial transcriptional landscape of human gastrulation and early brain development. Cell Stem Cell 30, 851–866.e7 (2023). 37192616 10.1016/j.stem.2023.04.016 PMC10241223

[21] H. H. Simon, H. Saueressig, W. Wurst, M. D. Goulding, D. D. O’Leary, Fate of midbrain dopaminergic neurons controlled by the engrailed genes. J. Neurosci. 21, 3126–3134 (2001). 11312297 10.1523/JNEUROSCI.21-09-03126.2001 PMC6762576

[22] M. T. Alves dos Santos, M. P. Smidt, En1 and Wnt signaling in midbrain dopaminergic neuronal development. Neural Dev. 6, 23 (2011). 21569278 10.1186/1749-8104-6-23 PMC3104484

[23] J. V. Veenvliet, M. T. M. Alves Dos Santos, W. M. Kouwenhoven, L. von Oerthel, J. L. Lim, A. J. A. van der Linden, M. J. A. Groot Koerkamp, F. C. P. Holstege, M. P. Smidt, Specification of dopaminergic subsets involves interplay of En1 and Pitx3. Development 140, 3373–3384 (2013). 23863478 10.1242/dev.094565

[24] G. A. Basile, M. Quartu, S. Bertino, M. P. Serra, M. Boi, A. Bramanti, G. P. Anastasi, D. Milardi, A. Cacciola, Red nucleus structure and function: From anatomy to clinical neurosciences. Brain Struct. Funct. 226, 69–91 (2021). 33180142 10.1007/s00429-020-02171-x PMC7817566

[25] A. Emmi, A. Antonini, V. Macchi, A. Porzionato, R. De Caro, Anatomy and connectivity of the subthalamic nucleus in humans and non-human primates. Front. Neuroanat. 14, 13 (2020). 32390807 10.3389/fnana.2020.00013 PMC7189217

[26] C. H. Asbreuk, C. F. Vogelaar, A. Hellemons, M. P. Smidt, J. P. Burbach, CNS expression pattern of Lmx1b and coexpression with ptx genes suggest functional cooperativity in the development of forebrain motor control systems. Mol. Cell. Neurosci. 21, 410–420 (2002). 12498783 10.1006/mcne.2002.1182

[27] N. Kee, N. Volakakis, A. Kirkeby, L. Dahl, H. Storvall, S. Nolbrant, L. Lahti, A. K. Bjorklund, L. Gillberg, E. Joodmardi, R. Sandberg, M. Parmar, T. Perlmann, Single-cell analysis reveals a close relationship between differentiating dopamine and subthalamic nucleus neuronal lineages. Cell Stem Cell 20, 29–40 (2017). 28094018 10.1016/j.stem.2016.10.003

[28] M. A. Cooper, K. Kobayashi, R. Zhou, Ephrin-A5 regulates the formation of the ascending midbrain dopaminergic pathways. Dev. Neurobiol. 69, 36–46 (2009). 19003794 10.1002/dneu.20685 PMC4026181

[29] P. S. Lo, V. V. Rymar, T. E. Kennedy, A. F. Sadikot, The netrin-1 receptor DCC promotes the survival of a subpopulation of midbrain dopaminergic neurons: Relevance for ageing and Parkinson’s disease. J. Neurochem. 161, 254–265 (2022). 35118677 10.1111/jnc.15579 PMC9305203

[30] S. Brignani, D. D. A. Raj, E. R. E. Schmidt, O. Dudukcu, Y. Adolfs, A. A. De Ruiter, M. Rybiczka-Tesulov, M. G. Verhagen, C. van der Meer, M. H. Broekhoven, J. A. Moreno-Bravo, L. M. Grossouw, E. Dumontier, J. F. Cloutier, A. Chedotal, R. J. Pasterkamp, Remotely produced and axon-derived netrin-1 instructs GABAergic neuron migration and dopaminergic substantia nigra development. Neuron 107, 684–702.e9 (2020). 32562661 10.1016/j.neuron.2020.05.037

[31] K. Tiklova, S. Nolbrant, A. Fiorenzano, A. K. Bjorklund, Y. Sharma, A. Heuer, L. Gillberg, D. B. Hoban, T. Cardoso, A. F. Adler, M. Birtele, H. Lunden-Miguel, N. Volakakis, A. Kirkeby, T. Perlmann, M. Parmar, Single cell transcriptomics identifies stem cell-derived graft composition in a model of Parkinson’s disease. Nat. Commun. 11, 2434 (2020). 32415072 10.1038/s41467-020-16225-5 PMC7229159

[32] H. J. R. Fernandes, N. Patikas, S. Foskolou, S. F. Field, J. E. Park, M. L. Byrne, A. R. Bassett, E. Metzakopian, Single-cell transcriptomics of Parkinson’s disease human in vitro models reveals dopamine neuron-specific stress responses. Cell Rep. 33, 108263 (2020). 33053338 10.1016/j.celrep.2020.108263

[33] J. Jerber, D. D. Seaton, A. S. E. Cuomo, N. Kumasaka, J. Haldane, J. Steer, M. Patel, D. Pearce, M. Andersson, M. J. Bonder, E. Mountjoy, M. Ghoussaini, M. A. Lancaster, H. S. Consortium, J. C. Marioni, F. T. Merkle, D. J. Gaffney, O. Stegle, Population-scale single-cell RNA-seq profiling across dopaminergic neuron differentiation. Nat. Genet. 53, 304–312 (2021). 33664506 10.1038/s41588-021-00801-6 PMC7610897

[34] A. Fiorenzano, E. Sozzi, M. Birtele, J. Kajtez, J. Giacomoni, F. Nilsson, A. Bruzelius, Y. Sharma, Y. Zhang, B. Mattsson, J. Emneus, D. R. Ottosson, P. Storm, M. Parmar, Single-cell transcriptomics captures features of human midbrain development and dopamine neuron diversity in brain organoids. Nat. Commun. 12, 7302 (2021). 34911939 10.1038/s41467-021-27464-5 PMC8674361

[35] G. Novak, D. Kyriakis, K. Grzyb, M. Bernini, S. Rodius, G. Dittmar, S. Finkbeiner, A. Skupin, Single-cell transcriptomics of human iPSC differentiation dynamics reveal a core molecular network of Parkinson’s disease. Commun. Biol. 5, 49 (2022). 35027645 10.1038/s42003-021-02973-7 PMC8758783

[36] A. Zagare, K. Barmpa, S. Smajic, L. M. Smits, K. Grzyb, A. Grunewald, A. Skupin, S. L. Nickels, J. C. Schwamborn, Midbrain organoids mimic early embryonic neurodevelopment and recapitulate LRRK2-p.Gly2019Ser-associated gene expression. Am. J. Hum. Genet. 109, 311–327 (2022). 35077669 10.1016/j.ajhg.2021.12.009 PMC8874228

[37] G. S. Virdi, M. L. Choi, J. R. Evans, Z. Yao, D. Athauda, S. Strohbuecker, R. S. Nirujogi, A. I. Wernick, N. Pelegrina-Hidalgo, C. Leighton, R. S. Saleeb, O. Kopach, H. Alrashidi, D. Melandri, J. Perez-Lloret, P. R. Angelova, S. Sylantyev, S. Eaton, S. Heales, D. A. Rusakov, D. R. Alessi, T. Kunath, M. H. Horrocks, A. Y. Abramov, R. Patani, S. Gandhi, Protein aggregation and calcium dysregulation are hallmarks of familial Parkinson’s disease in midbrain dopaminergic neurons. NPJ Parkinsons Dis. 8, 162 (2022). 36424392 10.1038/s41531-022-00423-7 PMC9691718

[38] P. Xu, H. He, Q. Gao, Y. Zhou, Z. Wu, X. Zhang, L. Sun, G. Hu, Q. Guan, Z. You, X. Zhang, W. Zheng, M. Xiong, Y. Chen, Human midbrain dopaminergic neuronal differentiation markers predict cell therapy outcomes in a Parkinson’s disease model. J. Clin. Invest. 132, e156768 (2022). 35700056 10.1172/JCI156768 PMC9282930

[39] Z. You, L. Wang, H. He, Z. Wu, X. Zhang, S. Xue, P. Xu, Y. Hong, M. Xiong, W. Wei, Y. Chen, Mapping of clonal lineages across developmental stages in human neural differentiation. Cell Stem Cell 30, 473–487.e9 (2023). 36933556 10.1016/j.stem.2023.02.007

[40] E. Bressan, X. Reed, V. Bansal, E. Hutchins, M. M. Cobb, M. G. Webb, E. Alsop, F. P. Grenn, A. Illarionova, N. Savytska, I. Violich, S. Broeer, N. Fernandes, R. Sivakumar, A. Beilina, K. J. Billingsley, J. Berghausen, C. B. Pantazis, V. Pitz, D. Patel, K. Daida, B. Meechoovet, R. Reiman, A. Courtright-Lim, A. Logemann, J. Antone, M. Barch, R. Kitchen, Y. Li, C. L. Dalgard, A. G. Center, P. Rizzu, D. G. Hernandez, B. E. Hjelm, M. Nalls, J. R. Gibbs, S. Finkbeiner, M. R. Cookson, K. Van Keuren-Jensen, D. W. Craig, A. B. Singleton, P. Heutink, C. Blauwendraat, The foundational data initiative for Parkinson disease: Enabling efficient translation from genetic maps to mechanism. Cell Genom. 3, 100261 (2023). 36950378 10.1016/j.xgen.2023.100261 PMC10025424

[41] D. Reumann, C. Krauditsch, M. Novatchkova, E. Sozzi, S. N. Wong, M. Zabolocki, M. Priouret, B. Doleschall, K. I. Ritzau-Reid, M. Piber, I. Morassut, C. Fieseler, A. Fiorenzano, M. M. Stevens, M. Zimmer, C. Bardy, M. Parmar, J. A. Knoblich, In vitro modeling of the human dopaminergic system using spatially arranged ventral midbrain-striatum-cortex assembloids. Nat. Methods 20, 2034–2047 (2023). 38052989 10.1038/s41592-023-02080-x PMC10703680

[42] H. S. Kim, Y. Xiao, X. Chen, S. He, J. Im, M. J. Willner, M. O. Finlayson, C. Xu, H. Zhu, S. J. Choi, E. V. Mosharov, H. W. Kim, B. Xu, K. W. Leong, Chronic opioid treatment arrests neurodevelopment and alters synaptic activity in human midbrain organoids. Adv. Sci. 11, e2400847 (2024). 10.1002/advs.202400847 PMC11151039 38549185

[43] J. Jo, Y. Xiao, A. X. Sun, E. Cukuroglu, H. D. Tran, J. Goke, Z. Y. Tan, T. Y. Saw, C. P. Tan, H. Lokman, Y. Lee, D. Kim, H. S. Ko, S. O. Kim, J. H. Park, N. J. Cho, T. M. Hyde, J. E. Kleinman, J. H. Shin, D. R. Weinberger, E. K. Tan, H. S. Je, H. H. Ng, Midbrain-like organoids from human pluripotent stem cells contain functional dopaminergic and neuromelanin-producing neurons. Cell Stem Cell 19, 248–257 (2016). 27476966 10.1016/j.stem.2016.07.005 PMC5510242

[44] X. Fan, Y. Fu, X. Zhou, L. Sun, M. Yang, M. Wang, R. Chen, Q. Wu, J. Yong, J. Dong, L. Wen, J. Qiao, X. Wang, F. Tang, Single-cell transcriptome analysis reveals cell lineage specification in temporal-spatial patterns in human cortical development. Sci. Adv. 6, eaaz2978 (2020). 32923614 10.1126/sciadv.aaz2978 PMC7450478

[45] Z. Luo, M. Xia, W. Shi, C. Zhao, J. Wang, D. Xin, X. Dong, Y. Xiong, F. Zhang, K. Berry, S. Ogurek, X. Liu, R. Rao, R. Xing, L. M. N. Wu, S. Cui, L. Xu, Y. Lin, W. Ma, S. Tian, Q. Xie, L. Zhang, M. Xin, X. Wang, F. Yue, H. Zheng, Y. Liu, C. B. Stevenson, P. de Blank, J. P. Perentesis, R. J. Gilbertson, H. Li, J. Ma, W. Zhou, M. D. Taylor, Q. R. Lu, Human fetal cerebellar cell atlas informs medulloblastoma origin and oncogenesis. Nature 612, 787–794 (2022). 36450980 10.1038/s41586-022-05487-2

[46] M. E. van der Heijden, R. V. Sillitoe, Interactions between Purkinje cells and granule cells coordinate the development of functional cerebellar circuits. Neuroscience 462, 4–21 (2021). 32554107 10.1016/j.neuroscience.2020.06.010 PMC7736359

[47] J. S. Fleck, F. Sanchis-Calleja, Z. He, M. Santel, M. J. Boyle, J. G. Camp, B. Treutlein, Resolving organoid brain region identities by mapping single-cell genomic data to reference atlases. Cell Stem Cell 28, 1177–1180 (2021). 34087154 10.1016/j.stem.2021.03.015

[48] H. Shi, Y. He, Y. Zhou, J. Huang, K. Maher, B. Wang, Z. Tang, S. Luo, P. Tan, M. Wu, Z. Lin, J. Ren, Y. Thapa, X. Tang, K. Y. Chan, B. E. Deverman, H. Shen, A. Liu, J. Liu, X. Wang, Spatial atlas of the mouse central nervous system at molecular resolution. Nature 622, 552–561 (2023). 37758947 10.1038/s41586-023-06569-5 PMC10709140

[49] C. L. Thompson, L. Ng, V. Menon, S. Martinez, C. K. Lee, K. Glattfelder, S. M. Sunkin, A. Henry, C. Lau, C. Dang, R. Garcia-Lopez, A. Martinez-Ferre, A. Pombero, J. L. R. Rubenstein, W. B. Wakeman, J. Hohmann, N. Dee, A. J. Sodt, R. Young, K. Smith, T. N. Nguyen, J. Kidney, L. Kuan, A. Jeromin, A. Kaykas, J. Miller, D. Page, G. Orta, A. Bernard, Z. Riley, S. Smith, P. Wohnoutka, M. J. Hawrylycz, L. Puelles, A. R. Jones, A high-resolution spatiotemporal atlas of gene expression of the developing mouse brain. Neuron 83, 309–323 (2014). 24952961 10.1016/j.neuron.2014.05.033 PMC4319559

[50] Z. Yao, C. T. J. van Velthoven, M. Kunst, M. Zhang, D. M. Millen, C. Lee, W. Jung, J. Goldy, A. Abdelhak, M. Aitken, K. Baker, P. Baker, E. Barkan, D. Bertagnolli, A. Bhandiwad, C. Bielstein, P. Bishwakarma, J. Campos, D. Carey, T. Casper, A. B. Chakka, R. Chakrabarty, S. Chavan, M. Chen, M. Clark, J. Close, K. Crichton, S. Daniel, P. D. Valentin, T. Dolbeare, L. Ellingwood, E. Fiabane, T. Fliss, J. Gee, J. Gerstenberger, A. Glandon, J. Gloe, J. Gould, J. Gray, N. Guilford, J. Guzman, D. Hirschstein, W. Ho, M. Hooper, M. Huang, M. Hupp, K. Jin, M. Kroll, K. Lathia, A. Leon, S. Li, B. Long, Z. Madigan, J. Malloy, J. Malone, Z. Maltzer, N. Martin, R. M. Cue, R. M. Ginty, N. Mei, J. Melchor, E. Meyerdierks, T. Mollenkopf, S. Moonsman, T. N. Nguyen, S. Otto, T. Pham, C. Rimorin, A. Ruiz, R. Sanchez, L. Sawyer, N. Shapovalova, N. Shepard, C. Slaughterbeck, J. Sulc, M. Tieu, A. Torkelson, H. Tung, N. V. Cuevas, S. Vance, K. Wadhwani, K. Ward, B. Levi, C. Farrell, R. Young, B. Staats, M.-Q. M. Wang, C. L. Thompson, S. Mufti, C. M. Pagan, L. Kruse, N. Dee, S. M. Sunkin, L. Esposito, M. J. Hawrylycz, J. Waters, L. Ng, K. Smith, B. Tasic, X. Zhuang, H. Zeng, A high-resolution transcriptomic and spatial atlas of cell types in the whole mouse brain. Nature 624, 317–332 (2023). 38092916 10.1038/s41586-023-06812-z PMC10719114

[51] L. Zhuo, M. Wang, T. Song, S. Zhong, B. Zeng, Z. Liu, X. Zhou, W. Wang, Q. Wu, S. He, X. Wang, MAPbrain: A multi-omics atlas of the primate brain. Nucleic Acids Res. 53, D1055–D1065 (2024). 10.1093/nar/gkae911 PMC11701655 39420633

[52] P. M. Almqvist, E. Akesson, L. U. Wahlberg, H. Pschera, A. Seiger, E. Sundstrom, First trimester development of the human nigrostriatal dopamine system. Exp. Neurol. 139, 227–237 (1996). 8654525 10.1006/exnr.1996.0096

[53] H. Lagercrantz, T. Ringstedt, Organization of the neuronal circuits in the central nervous system during development. Acta Paediatr. 90, 707–715 (2001). 11519969

[54] E. Vivi, B. Di Benedetto, Brain stars take the lead during critical periods of early postnatal brain development: Relevance of astrocytes in health and mental disorders. Mol. Psychiatry 29, 2821–2833 (2024). 38553540 10.1038/s41380-024-02534-4 PMC11420093

[55] T. Stuart, A. Butler, P. Hoffman, C. Hafemeister, E. Papalexi, W. M. Mauck III, Y. Hao, M. Stoeckius, P. Smibert, R. Satija, Comprehensive integration of single-cell data. Cell 177, 1888–1902.e21 (2019). 31178118 10.1016/j.cell.2019.05.031 PMC6687398

[56] C. Trapnell, D. Cacchiarelli, J. Grimsby, P. Pokharel, S. Li, M. Morse, N. J. Lennon, K. J. Livak, T. S. Mikkelsen, J. L. Rinn, The dynamics and regulators of cell fate decisions are revealed by pseudotemporal ordering of single cells. Nat. Biotechnol. 32, 381–386 (2014). 24658644 10.1038/nbt.2859 PMC4122333

[57] S. Jin, C. F. Guerrero-Juarez, L. Zhang, I. Chang, R. Ramos, C.-H. Kuan, P. Myung, M. V. Plikus, Q. Nie, Inference and analysis of cell-cell communication using CellChat. Nat. Commun. 12, 1088 (2021). 33597522 10.1038/s41467-021-21246-9 PMC7889871

[58] M. Efremova, M. Vento-Tormo, S. A. Teichmann, R. Vento-Tormo, CellPhoneDB: Inferring cell-cell communication from combined expression of multi-subunit ligand-receptor complexes. Nat. Protoc. 15, 1484–1506 (2020). 32103204 10.1038/s41596-020-0292-x

