# Supplementary material for: BrainSTEM: A single-cell multiresolution fetal brain atlas reveals transcriptomic fidelity of human midbrain cultures
Source: Sci Adv. 2025 Oct 31;11(44):eadu7944. doi: 10.1126/sciadv.adu7944 (PMC12577693; doi:10.1126/sciadv.adu7944)
Supplement: Supplementary file 1 — Figs. S1 to S4 Legends for tables S1 to S6 [file sciadv.adu7944_sm.pdf]

Science Advances
AAAS

Supplementary Materials for

BrainSTEM: A single-cell multiresolution fetal brain atlas reveals transcriptomic fidelity of human midbrain cultures

Hilary S. Y. Toh *et al.*

Corresponding author: Alfred X. Sun, alfred.sun@duke-nus.edu.sg; John F. Ouyang, john.ouyang@duke-nus.edu.sg

Sci. Adv. **11**, eadu7944 (2025)
DOI: 10.1126/sciadv.adu7944

The PDF file includes:

Figs. S1 to S4
Legends for tables S1 to S6

Other Supplementary Material for this manuscript includes the following:

Tables S1 to S6

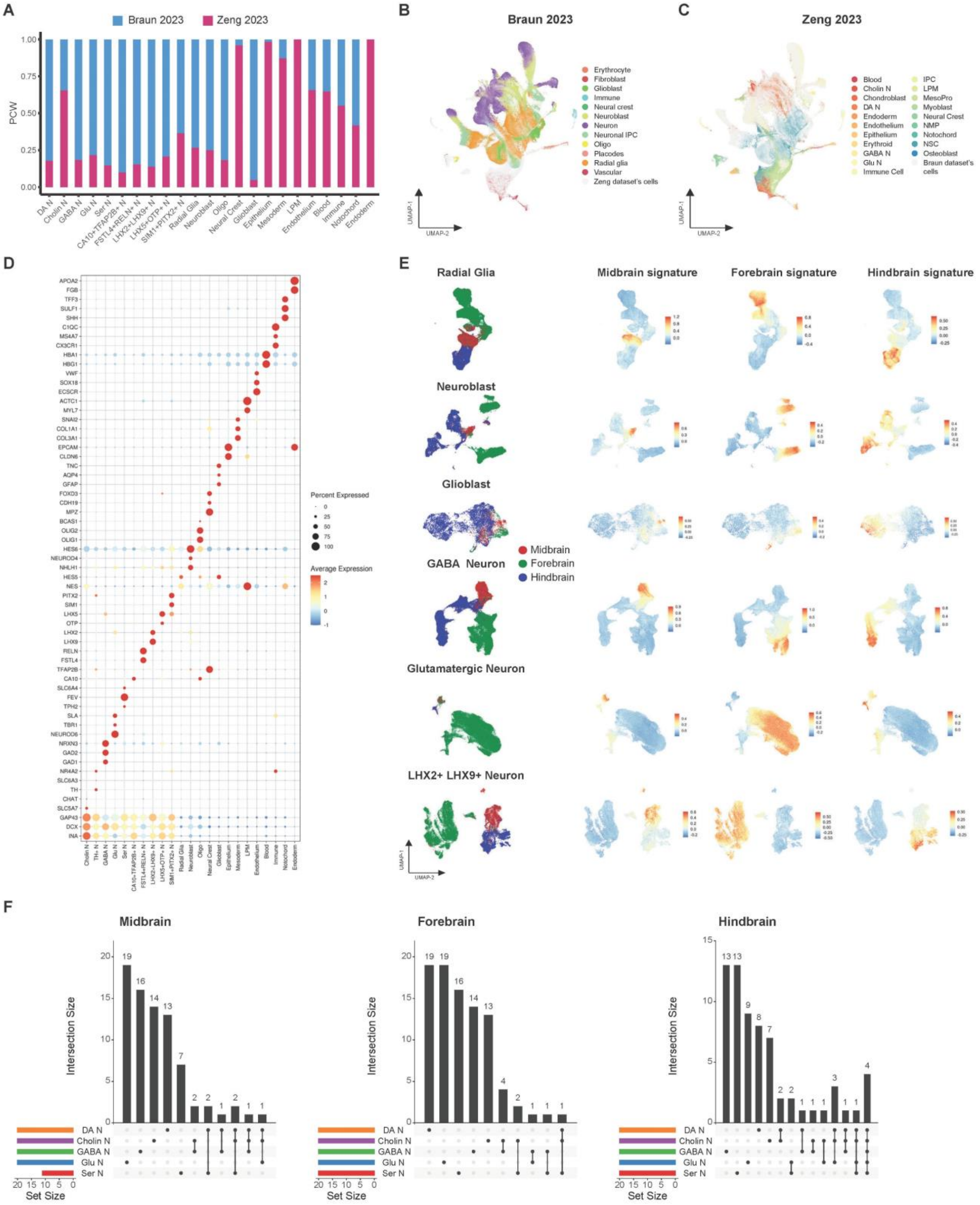
A
Braun 2023
Zeng 2023
PCW
DA N
Cholin N
GABA N
Glu N
Ser N
CA10+TFAP2B+ N
FSTL4+RELN+ N
LHX2+LHX9+ N
LHX5+OTP+ N
SIM1+PITX2+ N
Radial Glia
Neuroblast
Oligo
Neural Crest
Glioblast
Epithelium
Mesoderm
LPM
Endothelium
Blood
Immune
Notochord
Endoderm
B
Braun 2023
Erythrocyte
Fibroblast
Glioblast
Immune
Neural crest
Neuroblast
Neuron
Neuronal IPC
Oligo
Placodes
Radial glia
Vascular
Zeng dataset's cells
UMAP-1
UMAP-2
C
Zeng 2023
Blood
Cholin N
Chondroblast
DA N
Endoderm
Endothelium
Epithelium
Erythroid
GABA N
Glu N
Immune Cell
IPC
LPM
MesoPro
Myoblast
Neural Crest
NMP
Notochord
NSC
Osteoblast
Braun dataset's cells
D
Percent Expressed
Average Expression
E
Radial Glia
Midbrain signature
Forebrain signature
Hindbrain signature
Neuroblast
Glioblast
GABA Neuron
Midbrain
Forebrain
Hindbrain
Glutamatergic Neuron
LHX2+ LHX9+ Neuron
F
Midbrain
Forebrain
Hindbrain
Intersection Size
Set Size
DA N
Cholin N
GABA N
Glu N
Ser N

Fig. S1: Additional plots related to the construction of the fetal whole-brain single-cell atlas. (A) Proportion plot showing the proportion of cells coming from each reference single-cell study for each cell type. UMAP plot of fetal whole-brain atlas showing the original cell type annotation from (B) Braun *et al*. (*19*) and (C) Zeng *et al*. (*20*) (D) Dotplot of gene expression of full marker gene lists across different annotated cell types in the fetal whole-brain atlas, related to main Figure 1B. (E) UMAP plot of different region specific neural cell types showing the brain region where the cells are collected as well as the midbrain / forebrain / hindbrain region gene expression signature, related to main Figure 1I and 1J. (F) UpSet plots showing the distribution and overlap in midbrain / forebrain / hindbrain-region specific genes across five neuron classes (DA N, Cholin N, GABA N, Glu N, Ser N).

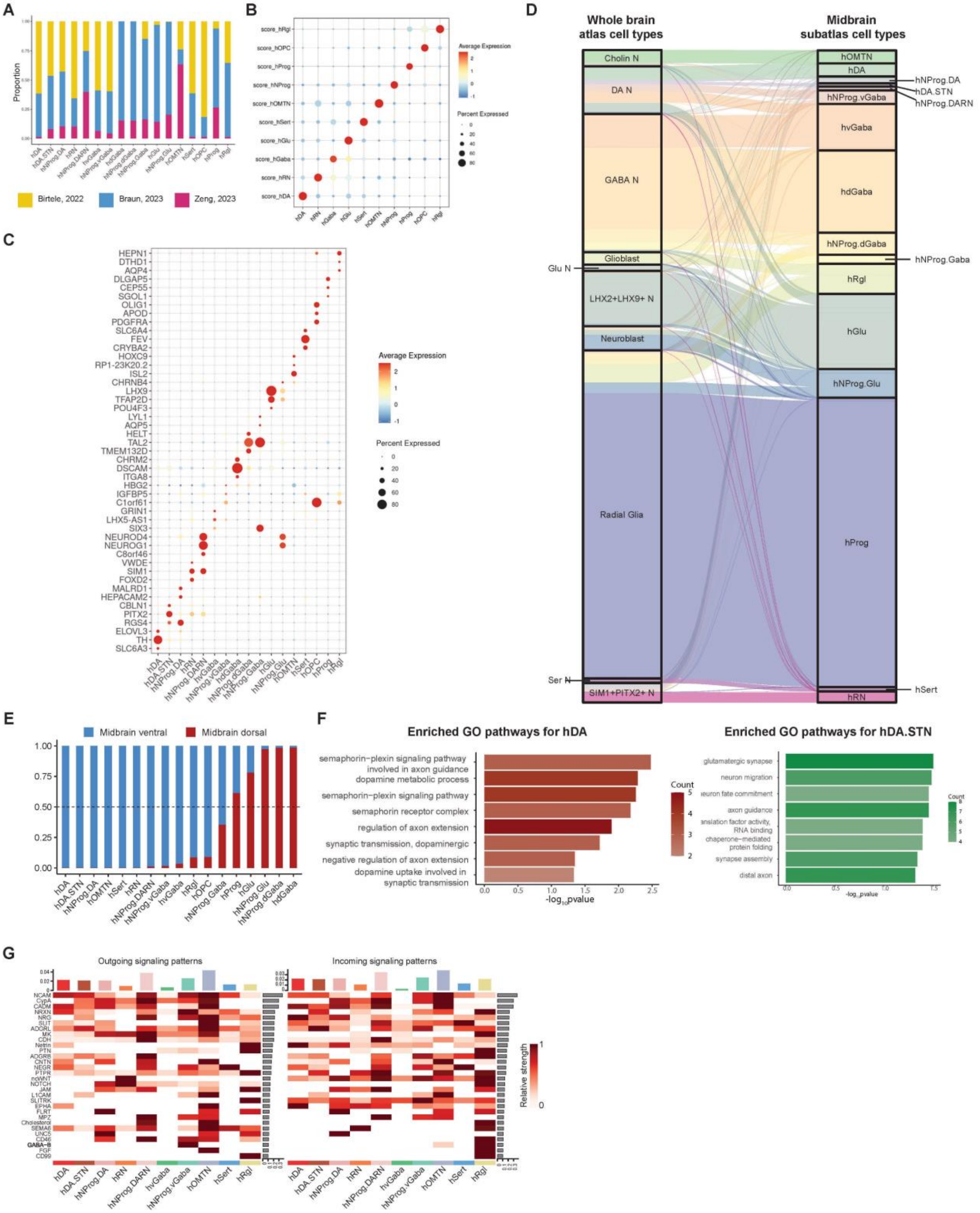

A
Proportion
hDA
hDA.STN
hNProg.DA
hRN
hNProg.DARN
hvGaba
hNProg.vGaba
hdGaba
hNProg.dGaba
hGlu
hNProg.Glu
hOMTN
hSert
hOPC
hProg
hRgl
Birtele, 2022
Braun, 2023
Zeng, 2023
B
score_hRgl
score_hOPC
score_hProg
score_hNProg
score_hOMTN
score_hSert
score_hGlu
score_hGaba
score_hRN
score_hDA
Average Expression
Percent Expressed
C
HEPN1
DTHD1
AQP4
DLGAP5
CEP55
SGOL1
OLIG1
APOD
PDGFRA
SLC6A4
FEV
CRYBA2
HOXC9
RP1-23K20.2
ISL2
CHRNB4
LHX9
TFAP2D
POU4F3
LYL1
AQP5
HELT
TAL2
TMEM132D
CHRM2
DSCAM
ITGA8
HBG2
IGFBP5
C1orf61
GRIN1
LHX5-AS1
SIX3
NEUROD4
NEUROG1
C8orf46
VWDE
SIM1
FOXD2
MALRD1
HEPACAM2
CBLN1
PITX2
RGS4
ELOVL3
TH
SLC6A3
Average Expression
Percent Expressed
D
Whole brain atlas cell types
Midbrain subatlas cell types
Cholin N
DA N
GABA N
Glioblast
Glu N
LHX2+LHX9+ N
Neuroblast
Radial Glia
Ser N
SIM1+PITX2+ N
hOMTN
hDA
hNProg.DA
hDA.STN
hNProg.DARN
hNProg.vGaba
hvGaba
hdGaba
hNProg.dGaba
hNProg.Gaba
hRgl
hGlu
hNProg.Glu
hProg
hSert
hRN
E
Midbrain ventral
Midbrain dorsal
F
Enriched GO pathways for hDA
semaphorin−plexin signaling pathway involved in axon guidance
dopamine metabolic process
semaphorin−plexin signaling pathway
semaphorin receptor complex
regulation of axon extension
synaptic transmission, dopaminergic
negative regulation of axon extension
dopamine uptake involved in synaptic transmission
Count
$-\log_{10}$pvalue
Enriched GO pathways for hDA.STN
glutamatergic synapse
neuron migration
neuron fate commitment
axon guidance
translation factor activity, RNA binding
chaperone−mediated protein folding
synapse assembly
distal axon
Count
G
Outgoing signaling patterns
Incoming signaling patterns
NCAM
CypA
CADM
NRXN
NRG
SLIT
ADGRL
MK
CDH
Netrin
PTN
ADGRB
CNTN
NEGR
PTPR
ncWNT
NOTCH
JAM
L1CAM
SLITRK
EPHA
FLRT
MPZ
Cholesterol
SEMA6
UNC5
CD46
GABA−B
FGF
CD99
Relative strength

Fig. S2: Additional plots related to the construction of the fetal midbrain subatlas. (A) Proportion plot showing the proportion of cells coming from each reference single-cell study for each midbrain cell type. Dotplot showing (B) the average module score across each midbrain cell type and (C) gene expression of full marker gene lists across different midbrain cell types. (D) Alluvial plot showing the concordance in annotation from whole brain cell type to midbrain cell type. (E) Proportion plot showing the proportion of cells from different midbrain subregions across different midbrain cell types. (F) Functional analysis of Gene Ontology pathways associated with differentially expressed genes between hDA and hDA.STN cell type. (G) Heatmap of outgoing and incoming signaling patterns from cell-cell communication analysis, across different midbrain cell types.

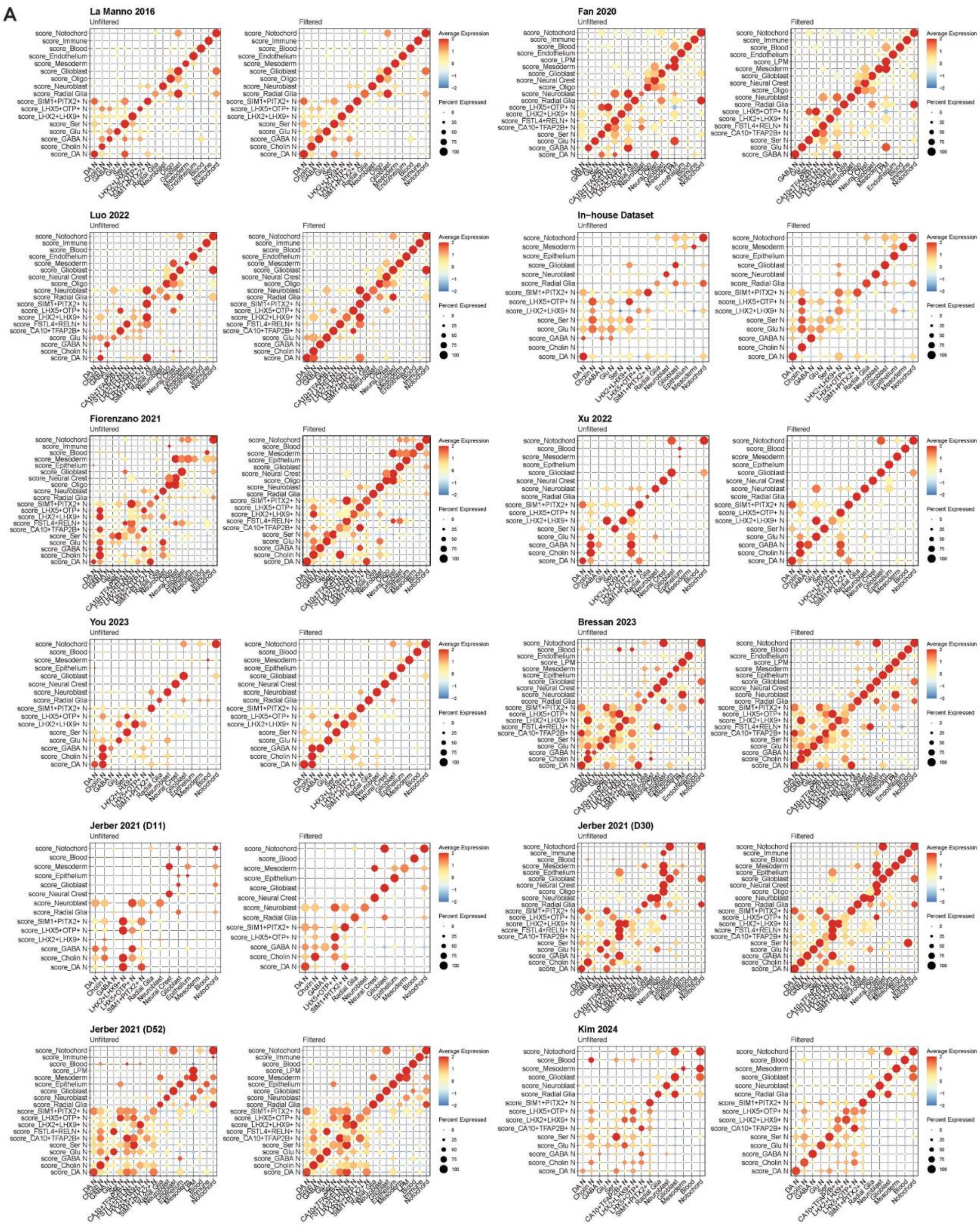
A
La Manno 2016
Fan 2020
Luo 2022
In−house Dataset
Fiorenzano 2021
Xu 2022
You 2023
Bressan 2023
Jerber 2021 (D11)
Jerber 2021 (D30)
Jerber 2021 (D52)
Kim 2024
Unfiltered
Filtered
Average Expression
Percent Expressed

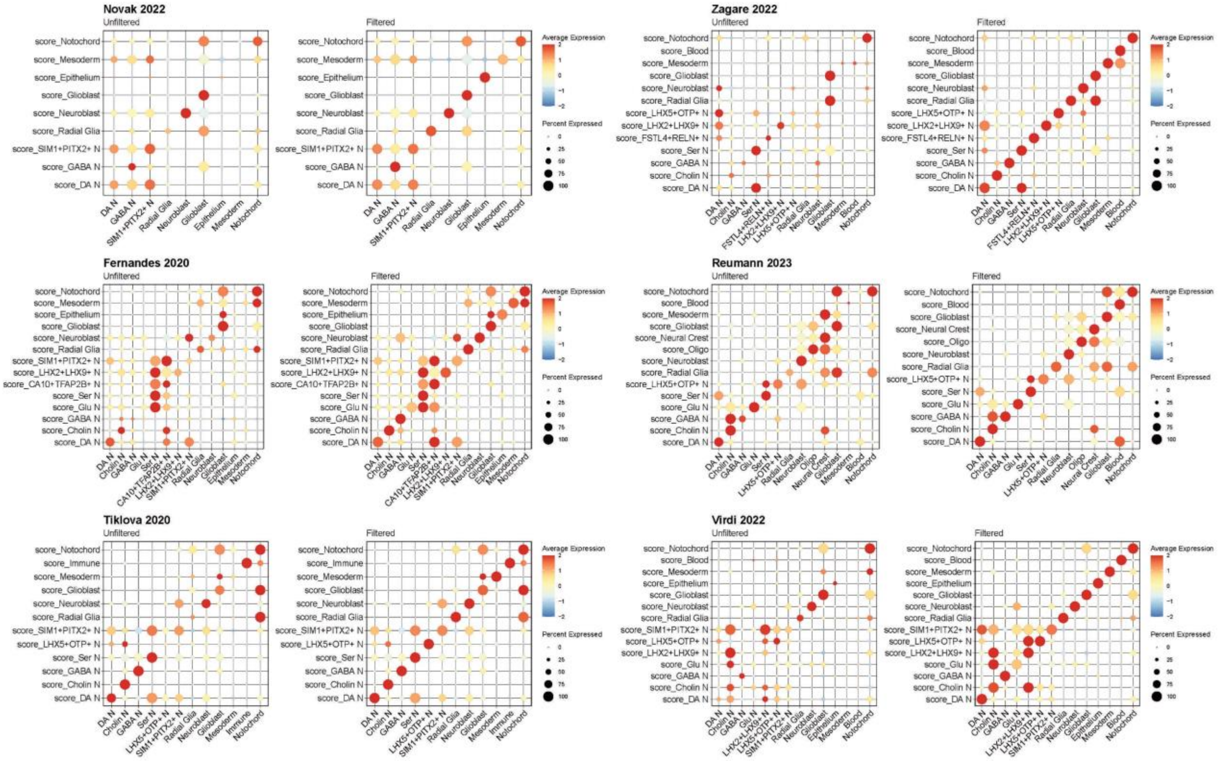

B

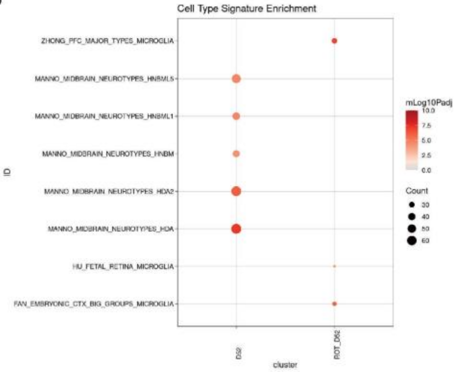

Fig. S3: Additional plots related to the projection of midbrain culture datasets onto the fetal whole brain atlas. (A) Series of dotplots before module score filtering (left panel) and after module score filtering (right panel) across predicted cell types for each of the projected *in vivo* and *in vitro*-derived datasets. (B) Gene oncology (GO) enrichment analysis on predicted hDA cells from the Jerber *et al.* (*33*) dataset.

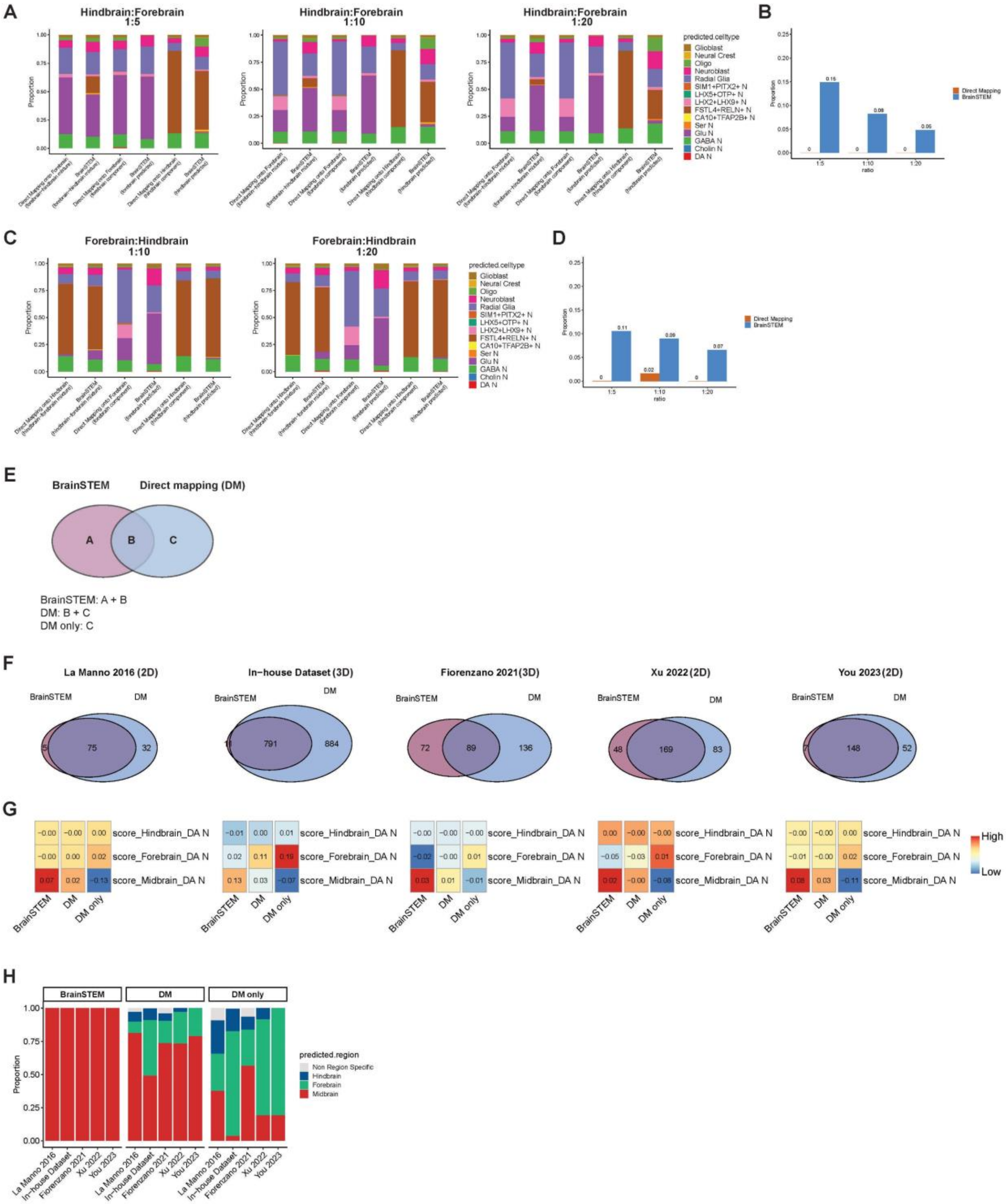

A
Hindbrain:Forebrain 1:5
Hindbrain:Forebrain 1:10
Hindbrain:Forebrain 1:20
Proportion
predicted.celltype
Glioblast
Neural Crest
Oligo
Neuroblast
Radial Glia
SIM1+PITX2+ N
LHX5+OTP+ N
LHX2+LHX9+ N
FSTL4+RELN+ N
CA10+TFAP2B+ N
Ser N
Glu N
GABA N
Cholin N
DA N
B
Direct Mapping
BrainSTEM
Proportion
ratio
1:5
1:10
1:20
0.15
0.08
0.05
C
Forebrain:Hindbrain 1:10
Forebrain:Hindbrain 1:20
D
0.11
0.02
0.09
0.07
E
BrainSTEM
Direct mapping (DM)
A
B
C
BrainSTEM: A + B
DM: B + C
DM only: C
F
La Manno 2016 (2D)
In-house Dataset (3D)
Fiorenzano 2021 (3D)
Xu 2022 (2D)
You 2023 (2D)
75
32
791
884
72
89
136
48
169
83
148
52
G
score_Hindbrain_DA N
score_Forebrain_DA N
score_Midbrain_DA N
BrainSTEM
DM
DM only
High
Low
H
predicted.region
Non Region Specific
Hindbrain
Forebrain
Midbrain
La Manno 2016
In-house Dataset
Fiorenzano 2021
Xu 2022
You 2023

Fig. S4: Plots demonstrating the advantages of the BrainSTEM framework over direct mapping. (A) Cell type proportions of synthetic dataset consisting of 1:5, 1:10 and 1:20 hindbrain to forebrain cells projected either by BrainSTEM or by DM. (B) Bar chart showing the proportion of FSTL4+ RELN+ N detected in synthetic datasets predicted by BrainSTEM or by DM. (C) Cell type proportions of synthetic dataset consisting of 1:5, 1:10 and 1:20 forebrain to hindbrain cells projected either by BrainSTEM or by DM. (D) Bar chart showing the proportion of Glu N detected in synthetic datasets predicted by BrainSTEM or by DM. (E) Schematic Venn diagram illustrating the definition of BrainSTEM, DM and DM only labels shown in Supplementary Figure 4C. (F) Venn diagrams showing the overlap of DA neurons predicted using BrainSTEM framework and DM for *in vivo* midbrain and selected *in vitro* differentiation datasets. (G) Brain region gene expression signatures calculated for DA neurons predicted using BrainSTEM, DM and cells uniquely called from direct mapping (DM only) for *in vivo* midbrain and different *in vitro* differentiation datasets. (H) Proportion plot showing the predicted brain region for *in vivo* midbrain and different *in vitro* differentiation datasets.

Table S1: Embryo sample information and brain region-cell type classification, related to Fig. 1 and S1.

Table S2: DEGs for cell types in fetal whole brain atlas, related to Fig. 1.

Table S3: DEGs for region-associated cell types in fetal whole brain atlas, related to Fig. 1 and S1.

Table S4: Fetal midbrain subatlas metadata and concordance of cell type labels between fetal whole brain atlas and fetal midbrain subatlas, related to Fig. 2.

Table S5: DEGs for cell types in fetal midbrain subatlas, for cell types in ventral midbrain subset and between hDA and hDA.STN cell types, related to Fig. 2.

Table S6: Publication details, cell numbers after each tier projection and reagents used in the midbrain differentiation protocols profiled in this study, related to Fig. 3, 4, S3 and S4.
